# Implications of Endocrine-Disrupting Chemicals for Human Health and Effective Methods for Prevention and Reduction

**DOI:** 10.3390/toxics14060515

**Published:** 2026-06-12

**Authors:** Codruța-Claudia Gherman-Lencu, Teodora-Gabriela Alexescu, Cristian Mureșanu, Cezara Andreea Gerdanovics, Mircea-Vasile Milaciu, Dana-Monica Iancu

**Affiliations:** 1 Endocrinology, Fundamental Discipline, Department 1, Iuliu Hațieganu University of Medicine and Pharmacy, Victor Babeș Street, Nr. 8, 400347 Cluj-Napoca, Romania; codruta_lmc@yahoo.com; 2 Department of Internal Medicine, 4th Medical Discipline, Iuliu Hațieganu University of Medicine and Pharmacy, Republicii Street, No. 18, 400015 Cluj-Napoca, Romania; andreea.ceza.irimie@elearn.umfcluj.ro (C.A.G.); vasile.milaciu@umfcluj.ro (M.-V.M.); 3 Laboratory of Molecular and Biomolecular Complexes, Department of Molecular and Isotopic Technologies, National Institute for Research and Development of Isotopic and Molecular Technologies, Donat Street, Nr. 67-103, 400293 Cluj-Napoca, Romania; cristim23@gmail.com; 4 Anatomy, Fundamental Discipline, Department 1, Iuliu Hațieganu University of Medicine and Pharmacy, Victor Babeș Street, Nr. 8, 400347 Cluj-Napoca, Romania; dr.danamonica@gmail.com

**Keywords:** endocrine-disrupting chemicals (EDCs), metabolism, genetic, epigenetic, obsesity, hormones, bisphenol A, prevention

## Abstract

Endocrine-disrupting chemicals (EDCs) are a heterogeneous group of exogenous compounds capable of interfering with hormonal homeostasis and endocrine-regulated physiological processes. Their widespread occurrence in food, water, air, consumer products and industrial materials has raised increasing concern regarding their contribution to chronic disease burden. This review synthesizes current evidence on the exposure characteristics, molecular mechanisms, health effects, and prevention strategies related to major EDC classes, including bisphenol A and phthalates, dioxins and polychlorinated biphenyls, per- and polyfluoroalkyl substances, pesticides, and brominated flame retardants. Evidence indicates that EDCs may act through receptor-mediated signaling, altered hormone synthesis and metabolism, oxidative stress, mitochondrial dysfunction, immune modulation, and epigenetic mechanisms, with effects that may vary according to dose, timing, sex, age, and developmental susceptibility. Reported health outcomes include metabolic and cardiovascular disorders, reproductive dysfunction, hormone-dependent cancers, thyroid disruption, immune dysregulation, and adverse developmental effects. Although complete avoidance is unrealistic, exposure reduction and risk mitigation can be achieved through coordinated individual, clinical, environmental, and regulatory interventions. A life-course approach is essential to limit the health burden associated with endocrine disruption.

## 1. Introduction

Endocrine-disrupting chemicals (EDCs) have a substantial potential to adversely affect the health of both humans [1] and wildlife [2].

Documented outcomes include reproductive disorders [3], neurobehavioral impairments in children, particularly associated with Bisphenol A (BPA) and phthalates, increased adiposity [4], and elevated risks of cancer, obesity, diabetes, and cardiovascular disease [5]. Some EDCs are classified as carcinogenic [6] or obesogenic [7], reflecting their capacity to disrupt metabolic and hormonal homeostasis. Given the diversity of sources and exposure pathways, it is essential to elucidate the role of EDCs in the onset and progression of these diseases.

EDCs are natural or synthetic substances that interfere with the normal functioning of the endocrine system [8]. They may mimic endogenous hormones (acting as agonistic EDCs) [9], block their actions (acting as antagonistic EDCs), or disrupt hormonal synthesis, release, transport, metabolism, receptor binding, or elimination [10]. In addition, EDCs can modify gene expression [11], influence enzymatic activity involved in hormone metabolism, and disturb the regulatory feedback mechanisms of the endocrine system. Consequently, EDCs impair hormonal homeostasis and may lead to a wide range of adverse health effects.

EDCs were formally described by the United States Environmental Protection Agency as exogenous agents that may interfere with the synthesis, secretion, transport, binding, action or elimination of endogenous hormones involved in homeostasis, reproduction, development and behavior. The Endocrine Society later broadened this concept by defining an endocrine disruptor as an exogenous chemical, or mixture of chemicals, capable of interfering with any aspect of hormone action [1].

EDCs include a wide range of natural and synthetic compounds, such as pesticides, fungicides, industrial chemicals, plasticizers, bisphenols, phthalates, nonylphenols, persistent organic pollutants, dioxins and dioxin-like compounds, polychlorinated biphenyls, brominated flame retardants, per- and polyfluoroalkyl substances, heavy metals, pharmaceutical agents and phytoestrogens. Natural EDCs include plant-derived estrogens (phytoestrogens), fungal estrogens (mycoestrogens), and naturally occurring toxic elements such as lead, mercury, and cadmium (lead, cadmium, and mercury are naturally occurring metals, but most human exposure occurs as a consequence of human activities). Synthetic EDCs comprise a broad spectrum of substances, including plastics and food packaging materials, pesticides and herbicides, flame retardants, industrial chemicals and their by-products, as well as ingredients commonly used in personal care products [1].

The large-scale production and use of synthetic chemicals over recent decades has substantially increased the number of substances released into the environment, many of which may have endocrine-active properties. These compounds may enter the environment during manufacturing, agricultural application, product use, waste disposal, incineration, or degradation of consumer products. Human exposure occurs mainly through ingestion of contaminated food and water but also through inhalation of indoor and outdoor air pollutants, dermal absorption, occupational contact, and maternal–fetal transfer. EDCs are found in many everyday products, including plastic bottles, food packaging, metal food cans, detergents, toys, cosmetics, pesticides, flame retardants, textiles, furniture, and electronic equipment. Exposure during prenatal life, infancy, and childhood is of particular concern because endocrine signaling plays a central role in organogenesis, neurodevelopment, growth, metabolism, and reproductive maturation [1].

From a regulatory perspective, EDC management remains challenging because these chemicals differ considerably in persistence, bioaccumulation, exposure patterns, dose–response behavior, and mechanisms of action. Persistent organic pollutants, including several organochlorine pesticides, polychlorinated biphenyls, dioxins, and furans, have been restricted or banned under international frameworks such as the Stockholm Convention. However, despite regulatory measures, many persistent pollutants remain detectable in environmental matrices and human biological samples due to their long half-lives, lipophilicity, bioaccumulation in adipose tissue, and long-range environmental transport. In contrast, non-persistent EDCs such as bisphenol A and several phthalates are rapidly metabolized and excreted, but continuous daily exposure through food packaging, plastics, cosmetics, and household products maintains public health concern. Moreover, structurally related substitutes, including alternative bisphenols, may present similar toxicological profiles, emphasizing the need for careful evaluation before substitution [1].

Microplastics represent an additional and increasingly relevant exposure pathway, but their role in endocrine disruption requires precise interpretation. The endocrine-disrupting potential of microplastics may derive not only from the polymeric particles themselves, but also from plastic additives incorporated during manufacturing, such as bisphenols, phthalates, flame retardants, stabilizers, and plasticizers. In addition, microplastics may adsorb and transport environmental contaminants, including persistent organic pollutants, pesticides, polycyclic aromatic hydrocarbons, and metals. Therefore, microplastics should be considered both as potential sources and vectors of endocrine-active substances rather than being uniformly classified as endocrine disruptors independent of their chemical composition [1].

From a regulatory and public health perspective, EDC management cannot rely exclusively on individual exposure avoidance. Although personal measures may reduce contact with selected consumer products, exposure to EDCs is often multi-source, multi-pathway, and influenced by factors beyond individual control. Therefore, effective prevention also requires population-level strategies, including regulatory restriction of hazardous chemicals, safer chemical substitution, improved testing before market authorization, environmental and human biomonitoring, cleaner production, waste management, occupational protection, and public education. This approach is consistent with recent policy-oriented analyses emphasizing that individual and clinical interventions may be useful but are insufficient without regulatory action to reduce the production, release, and persistence of endocrine-active chemicals in the environment [7].

Several international and regional initiatives have addressed hazardous chemicals and EDCs from a policy perspective, including the United Nations Sustainable Development Goals, the Stockholm Convention on Persistent Organic Pollutants, the WHO Chemicals Road Map, and European and United States regulatory efforts aimed at improving chemical safety assessment and reducing exposure to hazardous substances. However, implementation remains heterogeneous across jurisdictions, and EDCs are still insufficiently integrated into prevention policies despite their potential contribution to chronic disease risk [1,7].

Given the heterogeneity of EDCs, this review focuses on the most relevant classes from a public health perspective, selected according to their widespread exposure, persistence or continuous human contact, endocrine activity, documented health effects and regulatory relevance. Specifically, we discuss bisphenols and phthalates, dioxins and polychlorinated biphenyls, per- and polyfluoroalkyl substances, pesticides, brominated flame retardants, selected pharmaceuticals, and heavy metals. For each class, we aim to summarize major exposure sources, mechanisms of endocrine disruption, adverse health pathways, sex- and age-specific vulnerabilities, current prevention and exposure-reduction strategies, and remaining knowledge gaps that may justify further research and improved regulatory management.

## 2. Materials and Methods

A structured narrative literature review was conducted using PubMed/MEDLINE and Web of Science. The search was designed to identify peer-reviewed evidence and authoritative scientific reports addressing endocrine-disrupting chemicals, their exposure sources, mechanisms of action, adverse health effects, prevention strategies and regulatory or public health relevance.

The search included combinations of the following terms: “endocrine-disrupting chemicals”, “endocrine disruptors”, “bisphenol A”, “phthalates”, “polychlorinated biphenyls”, “dioxins”, “persistent organic pollutants”, “PFAS”, “pesticides”, “brominated flame retardants”, “pharmaceuticals”, “heavy metals”, “microplastics”, “exposure”, “mechanisms”, “receptor signaling”, “epigenetics”, “metabolic disorders”, “cardiovascular disease”, “reproductive toxicity”, “infertility”, “thyroid dysfunction”, “immune dysfunction”, “hormone-dependent cancers”, “children”, “prenatal exposure”, “prevention”, “exposure reduction”, “regulation” and “public health policy”.

The review focused primarily on studies published between January 2010 and December 2025. This time window was selected to capture the most recent evidence regarding EDC mechanisms, human health effects, exposure reduction and regulatory management, while allowing the inclusion of older landmark studies or policy documents when they provided foundational definitions, regulatory context, or historically relevant evidence. Additional references were identified through manual screening of the reference lists of relevant reviews, systematic reviews, meta-analyses and reports issued by scientific or regulatory organizations.

The inclusion criteria were as follows: (1) original experimental, epidemiological, biomonitoring, occupational, cross-sectional, cohort, or mechanistic studies addressing EDC exposure or effects; (2) systematic reviews, meta-analyses, narrative reviews, consensus statements, and authoritative scientific or regulatory reports; (3) studies addressing major EDC classes or representative compounds, exposure pathways, molecular or adverse outcome mechanisms, human health effects, prevention strategies, or regulatory/public health management; and (4) articles published in English.

The exclusion criteria were as follows: (1) studies not relevant to endocrine disruption; (2) articles focused exclusively on wildlife or ecological outcomes without clear relevance to human health; (3) papers lacking sufficient methodological, toxicological, mechanistic, or clinical detail; (4) duplicate records; (5) conference abstracts, letters, editorials, and non-peer-reviewed sources unless they represented official regulatory or public health documents; and (6) articles not aligned with the aims of the review.

The literature selection process involved title and abstract screening, followed by full-text assessment of potentially relevant articles. A total of 412 records were initially identified. After removal of duplicates and exclusion of clearly irrelevant records, a total of 232 sources were included in the final narrative synthesis. These consisted of 71 original human studies, including epidemiological, biomonitoring, occupational and cross-sectional or cohort studies; 54 experimental or mechanistic studies, including in vitro, animal, and toxicological models; 73 reviews, including narrative, mechanistic, semi-structured, systematic reviews, and meta-analyses; and 34 policy documents, regulatory reports, guidelines, consensus statements, book chapters or authoritative scientific resources. The evidence was not pooled quantitatively because the included sources differed substantially in chemical class, exposure assessment, biological matrix, study population, outcome definition and methodological design.

Where available in the included literature, sex-specific and gender-relevant findings were extracted and discussed, particularly for reproductive outcomes, pregnancy, puberty, fertility, developmental vulnerability and hormone-dependent cancers. Because this review is based on previously published evidence, sex-disaggregated data were reported only when available in the original sources.

For synthesis, studies were grouped according to the main EDC class or exposure category: bisphenols and phthalates; dioxins and polychlorinated biphenyls; per- and polyfluoroalkyl substances and pesticides; brominated flame retardants; selected pharmaceuticals; heavy metals; and microplastics as potential vectors or sources of endocrine-active substances. Within each group, evidence was further organized according to exposure pathway, molecular mechanism or adverse outcome pathway, affected population, reported health outcomes, prevention strategies, and remaining knowledge gaps. When multiple studies addressed similar outcomes, their findings were compared qualitatively to identify convergent evidence, inconsistencies, methodological limitations, and areas requiring further research. Given the heterogeneity of chemical classes, exposure metrics, populations, and outcomes, a quantitative meta-analysis was not performed.

## 3. Results

### 3.1. Main Characteristics of Exposure to EDC’s

**Figure 1:**
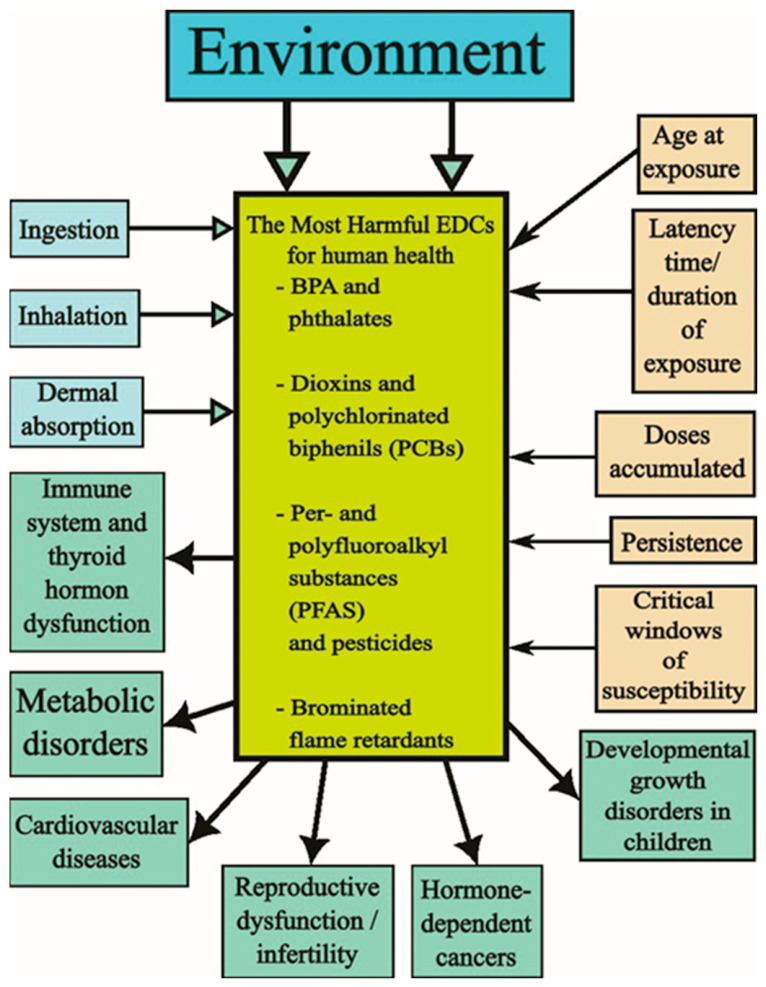
Schematic representation of the main characteristics of exposure to endocrine-disrupting chemicals (EDCs), including major exposure pathways, dose and timing-related factors, selected EDC classes of public health concern and major human health risks associated with EDC exposure. BPA, bisphenol A; BFRs, brominated flame retardants; PCBs, polychlorinated biphenyls; PFAS, per- and polyfluoroalkyl substances.

The main characteristics of exposure to EDCs encompass several interrelated factors, including the age at exposure, latency period, dose, persistence, exposure pathways, and mechanisms of action, as illustrated in Figure 1.

#### 3.1.1. Age at Exposure to EDC’s

Age at exposure is a critical determinant of the biological impact of EDCs, as fetuses and developing children are considerably more vulnerable than adults. The fetus, infant, and child exhibit increased sensitivity to environmental stressors such as EDCs due to their rapid developmental processes and proportionally greater exposure, which results from age-specific behaviors, anatomical features, and physiological characteristics [12].

Numerous studies have shown that elevated exposure to endocrine-disrupting compounds is associated with adverse reproductive outcomes and developmental abnormalities [13].

#### 3.1.2. Latency Time and Duration of Exposure to EDC’s

Latency time is a critical factor in EDC exposure, as adverse health effects may not manifest until years after the initial exposure [14]. In some instances, these effects can be transmitted multi- and transgenerational [15], with potentially increasing severity, due to the impact of EDCs on male and female fertility [16].

Both acute and delayed (latent) effects of EDCs have been documented [17]. The concept of time in this context encompasses the period over which an outcome is expected to occur, including both the timing and duration of exposure and the interval between exposure and the manifestation of health effects (latency) [18].

#### 3.1.3. Doses Accumulated

The interpretation of low-dose effects remains debated. Some studies and reviews suggest that EDCs may exert biologically relevant effects at very low levels of exposure, particularly because endocrine systems are sensitive to small hormonal perturbations and because some compounds may display non-monotonic dose–response relationships [19,20,21]. However, other risk assessment-oriented analyses have argued that the evidence for consistent low-dose effects and non-monotonic dose–response patterns is not sufficient for all EDCs and endpoints, emphasizing the need for compound-specific evaluation, reproducibility, biological plausibility, and clear distinction between adaptive biological responses and adverse outcomes [20].

Therefore, low-dose exposure should be interpreted cautiously, considering the specific chemical, exposure window, target tissue, endpoint assessed, and whether the observed biological change results in measurable functional impairment or increased disease risk. Small perturbations in hormone concentrations can have biologically significant consequences, highlighting the sensitivity of endocrine systems to minimal chemical disturbances [21,22,23].

#### 3.1.4. Persistence of EDC’s

Persistence is a key characteristic of several EDC classes, particularly persistent organic pollutants such as dioxins, PCBs, and certain organochlorine pesticides. Due to their lipophilic properties, these compounds can bioaccumulate in the food chain and in adipose tissue, remaining in the body for extended periods and contributing to prolonged internal exposure [1]. However, this characteristic does not apply uniformly to all EDCs, as non-persistent compounds such as BPA and several phthalates may be rapidly metabolized and excreted, while still raising concern because of continuous daily exposure [1]. They are present in the air we breathe, the food we consume, and the water we drink, and are also encountered in everyday products, including personal care items, household cleaning agents, furniture, and children’s toys [24].

#### 3.1.5. Critical Windows of Susceptibility

Exposures to EDCs during sensitive developmental stages, often referred to as critical windows of susceptibility, are of particular concern and warrant precise identification [25].

Disruption during these periods can predispose individuals to disease later in life, a concept originally described as the “fetal origins of adult disease” [26] hypothesis, popularized by Dr. David Barker [27], and now commonly termed the “Developmental Origins of Health and Disease” [28].

Barker proposed that adverse environmental exposures during fetal development, including nutritional deficiencies or maternal stress, can induce structural, metabolic [29], and epigenetic changes [30] that increase the risk of chronic diseases such as coronary heart disease, hypertension, obesity, and diabetes in adulthood.

### 3.2. Major Pathways of Exposure to EDC’s

The route of exposure—whether by ingestion, inhalation, or dermal absorption—significantly influences the toxicokinetics of EDCs and the overall risk to health [31]. Importantly, exposure route alone is insufficient to define toxicological or endocrine-disrupting risk. The biological relevance of exposure depends on dose, concentration at the target tissue, frequency and duration of exposure, toxicokinetics, bioavailability, persistence, metabolism, route-specific absorption, and the developmental window during which exposure occurs. This distinction is essential because the mere presence of a substance in food, water, air, or consumer products does not necessarily imply adverse endocrine activity at real-life exposure levels. Conversely, some endocrine-active chemicals may raise concern at relatively low doses when exposure occurs during sensitive windows, when exposure is chronic or cumulative, or when compounds act as mixtures. Therefore, in this review, exposure pathways are discussed together with timing, duration, persistence, and dose–response considerations, rather than as isolated routes of contact.

Figure 1 depicts a visual representation of the above-mentioned exposure pathways, along with the most significant human health risks associated with EDC exposure. EDCs can accumulate in various tissues and disrupt hormonal signaling through mechanisms including receptor binding, oxidative stress, and epigenetic modifications [32].

#### 3.2.1. Exposure Through Ingestion

Ingestion represents a major pathway of EDC exposure, as these chemicals tend to bioaccumulate within the food web, and microplastics can absorb and subsequently release harmful compounds [33]. Consumption of foodstuffs such as canned products, bottled water, dairy, fish, meat, eggs, and vegetables containing EDCs is considered one of the principal routes of human exposure to these products [34].

#### 3.2.2. Exposure Through Inhalation

Many EDCs are anthropogenic in origin, and inhalation of contaminated air constitutes an important exposure pathway. Although multiple exposure routes have been documented, receptor-mediated interactions can occur directly via inhalation [35], in addition to simultaneous absorption and ingestion [36]. Developmental exposure to common environmental pollutants has been shown to produce long-term disruptions of hormonal feedback regulation at the hypothalamic–pituitary level [37].

#### 3.2.3. Exposure Through Dermal Absorption

Dermal absorption [38] represents another significant pathway of EDC exposure, particularly through the use of cosmetics and personal hygiene products, which involve continuous and prolonged contact. Such exposure has been linked to reproductive dysfunction, cancer, and neurological disorders. In addition, EDCs can exhibit carcinogenic, immunotoxic, and hepatotoxic effects on human skin [39].

### 3.3. The Mechanistic Molecular Action of the EDC’s

The molecular mechanisms of EDCs are diverse, with different compounds interfering at multiple points within endocrine pathways. Key mechanistic categories include receptor-mediated signaling, gene regulation, enzymatic interference, and epigenetic modifications, including transgenerational epigenetic effects [15,16,40].

For example, BPA has been widely investigated as a xenoestrogen because of its ability to interact with estrogen receptors and other endocrine-related signaling pathways. However, the interpretation of BPA-related endocrine activity remains debated, as experimental, epidemiological, and regulatory assessments have not always reached consistent conclusions regarding the relevance of these effects at human exposure levels. Therefore, BPA should be discussed as a compound with reported endocrine activity in several experimental and observational settings, while acknowledging that risk assessment conclusions may differ according to study design, dose, endpoint, and regulatory evaluation framework [41]. EDCs can modulate both genomic and non-genomic estrogen receptor activity through multiple mechanisms: (a) direct binding to the ligand-binding pocket of estrogen receptors [42]; (b) indirect modulation via transcription factors [43], such as the aryl hydrocarbon receptor; and (c) alteration of metabolic enzymes essential for normal estrogen synthesis and metabolism [44]. Non-genomic signaling pathways include rapid activation of second messengers or kinases, further contributing to endocrine disruption [45].

EDCs exert their effects by altering endogenous hormone concentrations, modifying hormone availability, or affecting hormone receptor turnover [46]. In other instances, EDCs act downstream of receptor activation [47] by interacting with components of hormone signaling pathways rather than the receptors themselves. Some EDCs possess chemical structures that differ substantially from endogenous hormones; for example, fluoxetine, a selective serotonin reuptake inhibitor, has been shown to modify multiple intracellular signaling pathways in various cell types [46].

Beyond receptor interactions, EDCs can interfere with enzymes involved in steroidogenesis [48] and participate in epigenetic mechanisms with transgenerational consequences [15]. EDCs have been shown to promote epigenetic inheritance of disease and abnormal physiology, including programming of primordial germ cells that may contribute to transgenerational genetic and epigenetic alterations [49].

Early-life exposure to plastic-derived EDCs, such as BPA, bis(2-ethylhexyl) phthalate, and dibutyl phthalate [50], has been associated with adverse health outcomes later in life, including diabetes, obesity, cancer, reproductive disorders, and epigenetic transgenerational inheritance of obesity [51], reproductive disease, and sperm epimutations [52]. For instance, BPA exposure during early development has been linked to an increased risk of prostate cancer [53] in adulthood.

### 3.4. Associated Human Health Risks Posed by EDC’s

EDCs have been reported in association with a wide range of human health outcomes, including metabolic disorders, cardiovascular diseases, reproductive dysfunction and infertility, hormone-dependent cancers, developmental and growth abnormalities in children, and immune and thyroid hormone dysfunction. However, these associations should not be interpreted uniformly as evidence of causation, because the strength of evidence differs by compound, exposure level, biological mechanism, study design, population, and outcome. In many cases, causal inference remains limited by observational designs, mixture exposures, exposure misclassification, long latency periods, and residual confounding.

Because EDCs differ substantially in chemical structure, persistence, bioaccumulation, exposure route, and endocrine target, their health effects should not be interpreted as uniform class effects. Instead, the available evidence suggests partially distinct patterns according to chemical category, target population, and biological pathway. For example, bisphenols and phthalates are mainly discussed in relation to continuous exposure from plastics, food packaging, cosmetics, and consumer products, with particular relevance for pregnant women, children, and reproductive-age adults. Persistent organic pollutants, including dioxins, polychlorinated biphenyls, and organochlorine pesticides, are more strongly characterized by long-term persistence, lipophilicity, bioaccumulation, placental transfer, and effects on reproductive, metabolic, thyroid, immune and developmental endpoints. Other groups, such as PFAS, brominated flame retardants, selected pharmaceuticals, nonylphenols, and heavy metals, have been linked to more specific endocrine axes or vulnerable exposure settings, including occupational exposure, indoor dust exposure, prenatal development, and childhood growth [1]. Therefore, the main EDC classes, their representative compounds, target populations, mechanisms, and associated outcomes are summarized in Table 1.

This class-based organization also highlights that the strength and nature of evidence differ across EDC categories. Bisphenols and phthalates are frequently investigated in relation to reproductive and metabolic outcomes because exposure is continuous and widespread, despite their relatively rapid elimination. In contrast, dioxins, PCBs and organochlorine pesticides are particularly relevant for long-term and developmental outcomes because of their persistence, lipophilicity, bioaccumulation and capacity for placental and lactational transfer. For PFAS, brominated flame retardants, heavy metals, and pharmaceuticals, the evidence is more heterogeneous and often depends on specific exposure settings, such as contaminated water, indoor dust, occupational contact, or prenatal exposure. This distinction is important because prevention and monitoring strategies must be adapted to the chemical class, target population, and dominant route of exposure rather than being applied uniformly to all EDCs [1].

The comparative organization of the evidence indicates that some associations are more consistently supported than others. Several epidemiological studies, mechanistic investigations, systematic reviews, and meta-analyses converge on the involvement of bisphenols, phthalates, PCBs, dioxins, organochlorine pesticides, PFAS, brominated flame retardants, and selected metals in reproductive, metabolic, thyroid, developmental, immune, cardiovascular, and carcinogenic outcomes. The strongest evidence coverage in the present review was found for reproductive and developmental outcomes, followed by metabolic disorders, cancer-related endpoints, thyroid disruption, cardiovascular outcomes, and immune effects. By contrast, the evidence for microplastics, substitute bisphenols, mixed chemical exposures, selected pharmaceuticals, and some personal care-related EDCs remains more heterogeneous, partly because of differences in exposure biomarkers, biological matrices, study design, timing of exposure, population characteristics, and outcome definitions. Therefore, this synthesis emphasizes areas of convergence and uncertainty across EDC classes rather than interpreting all endocrine disruptors as a uniform exposure category. The descriptive evidence mapping of the literature included in the review, according to EDC class and main outcome domain, is presented in Table 2.

#### 3.4.1. Metabolic Disorders

**Figure 2:**
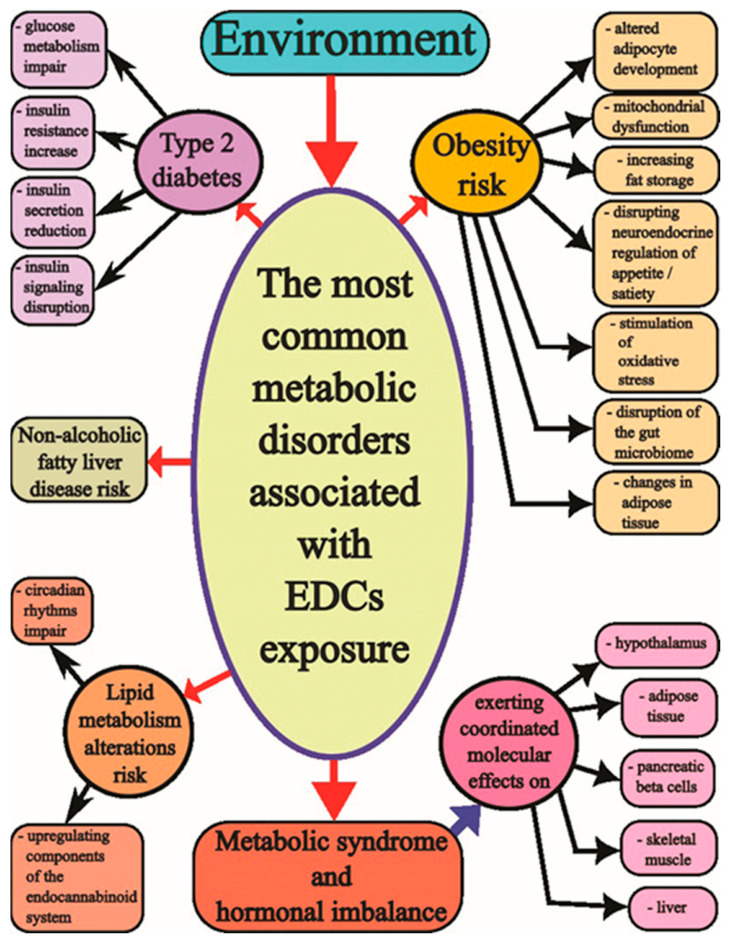
Schematic representation of the most common metabolic disorders, associated with EDCs exposure and their physiological, biological and biochemical effects.

Metabolic effects are among the most frequently reported outcomes of EDC exposure, particularly for bisphenols, phthalates, POPs, PCBs, organochlorine pesticides, PFAS and selected heavy metals. Figure 2 depicts a visual representation of the metabolic disorders, along with the most significant physiological and biochemical effects associated with EDC exposure.

Obesity is influenced by multiple factors, including exposure to obesogenic EDCs [54,55] such as bisphenols, phthalates, polychlorinated biphenyls, parabens, fluorinated chemicals, and pesticides.

These compounds may promote adiposity through altered adipocyte differentiation, PPARγ activation, mitochondrial dysfunction, oxidative stress, disruption of appetite and satiety signaling, gut microbiota changes and impaired thermogenic adipose tissue activity [56,57,58,59,60,61]. For type 2 diabetes, the most frequently implicated EDC classes include PCBs, dioxins, organochlorine pesticides, BPA, phthalates, PFAS, cadmium, and arsenic. These compounds may impair glucose metabolism through insulin resistance, altered insulin secretion, pancreatic β-cell toxicity, oxidative stress, and mitochondrial dysfunction [62,63,64,65]. Metabolic Syndrome and Hormonal Imbalance: EDCs can significantly perturb metabolic syndrome [66] and hormonal homeostasis by exerting coordinated molecular effects on multiple metabolically active organs, including the hypothalamus, adipose tissue, pancreatic beta cells, skeletal muscle, and liver [67].

Non-Alcoholic Fatty Liver Disease is strongly associated with environmental EDC exposure, including phthalates [68] (used in plastics, food packaging, and cosmetics), cadmium (used in batteries, pigments, plastic stabilizers, alloys, and coatings [50]), and BPA (found in food and beverage packaging).

Also, EDC exposure can disrupt lipid metabolism by affecting circadian rhythms and upregulating components of the endocannabinoid system, both of which contribute to increased hepatic lipid accumulation [69,70].

#### 3.4.2. Cardiovascular Diseases

Cardiovascular outcomes have been most frequently linked to metals, persistent organic pollutants, BPA, phthalates, and selected pesticide-related metabolites. Reported associations include cardiovascular mortality, endothelial dysfunction, atherosclerosis, and altered cardiometabolic risk profiles [71,72,73]. EDCs can elevate cardiovascular risk through several mechanisms, including epigenetic regulation and the transgenerational inheritance of cardiovascular disease susceptibility [74]. Although the precise pathways remain incompletely understood, certain exogenous environmental chemicals—such as BPA, nonylphenol, organochlorine pesticides, polychlorinated biphenyls, and phthalates—have been reported to increase cardiovascular risk [75].

Several EDCs that mimic or interfere with estrogen signaling are recognized for impairing vascular endothelial function. These chemicals interact with estrogen receptors, increase oxidative stress, and disrupt molecular pathways critical for vascular homeostasis. Notable examples include polychlorinated biphenyls [76], BPA [77], phthalates and other plastic additives [78], perinatal exposure to diethylstilbestrol [79], and persistent organic pollutants [80].

#### 3.4.3. Reproductive Dysfunction and Infertility

Reproductive outcomes show clear sex-specific patterns, with phthalates, BPA, PCBs, dioxins, organochlorine pesticides, and selected metals being the most frequently implicated classes. In men, EDCs can impair sperm motility, concentration, volume, and morphology, and increase sperm DNA damage [81].

In males, these effects are mainly related to altered testicular development, impaired steroidogenesis, anti-androgenic activity, androgen receptor modulation, and Leydig cell dysfunction [82,83,84,85]. Experimental studies in mice and rats have shown that exposure to BPA is associated with decreased testosterone levels [86], impaired sperm motility, increased DNA damage, reduced sperm counts [87], inhibition of sperm motion kinematics through ATP depletion in spermatozoa [88], disrupting spermatogenesis [89], and decreased epididymal sperm concentration, among other effects [90]. These findings illustrate the complex and potentially hazardous effects of BPA on male reproductive health.

In women, EDC exposure can lead to a range of reproductive abnormalities, including blocked fallopian tubes, ovarian disorders such as polycystic ovary syndrome, uterine dysfunction, impaired oocyte production, reduced oocyte quality, local inflammation, and broader endocrine disturbances [91].

Additional outcomes include endometriosis, premature ovarian failure, menstrual irregularities, altered menarche [13], and infertility [92].

Epidemiological evidence indicates that female reproductive health is particularly sensitive to estrogenic EDCs [93] present in pharmaceuticals, polychlorinated biphenyls, organochlorine pesticides [94], industrial products such as plasticizers, and phytoestrogens [95].

Animal studies further demonstrate that neonatal exposure to diethylstilbestrol can induce polyovular follicles in the ovaries [96] or promote ovary-independent vaginal stratification [97], highlighting the developmental susceptibility to EDCs.

It is important to note that not all phytoestrogens are harmful to human health. For example, genistein, a phytoestrogen derived from soy, has been associated with beneficial effects.

Epidemiological data indicate that increased lignan intake is correlated with a reduced incidence of uterine fibroids [98], consistent with other studies demonstrating a significant inverse relationship between the consumption of green vegetables and fruits and the prevalence of uterine fibroids [99].

Therefore, although animal studies suggest that certain EDC exposures can induce leiomyoma in adulthood, extrapolating these findings to human women and predicting a similar risk of uterine fibroids remains challenging.

#### 3.4.4. Hormone-Dependent Cancers

**Figure 3:**
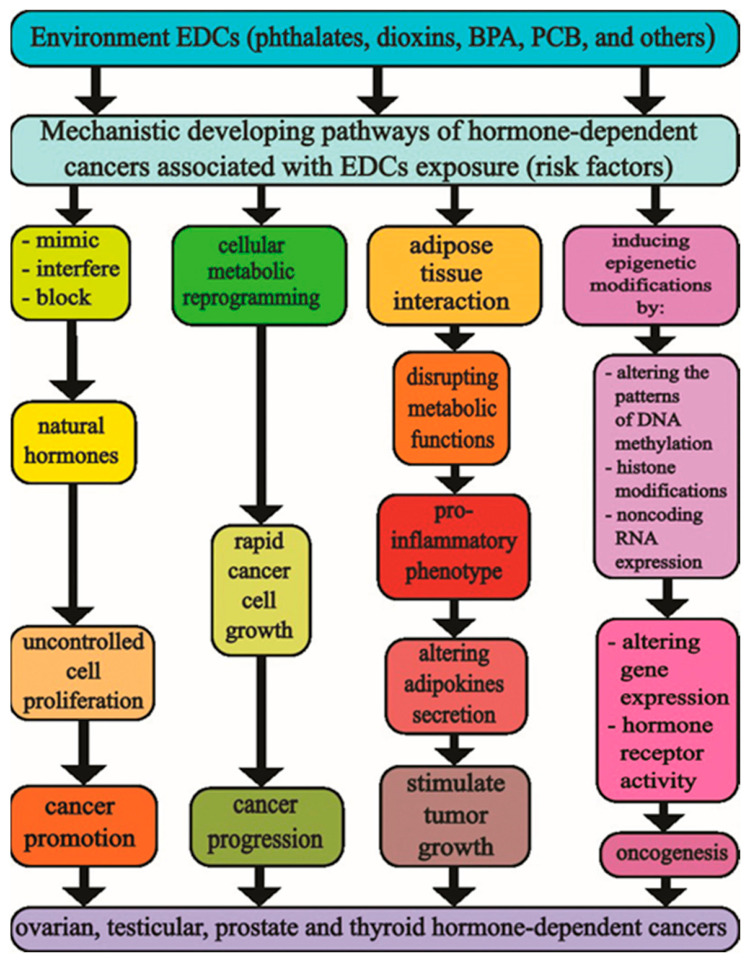
Schematic representation of the mechanistic pathways involved in hormone-dependent cancers associated with exposure to EDCs, and their physiological, biological, molecular, and biochemical effects.

Hormone-dependent cancers have been mainly discussed in relation to BPA, phthalates, dioxins, PCBs, organochlorine pesticides, and selected estrogenic or anti-estrogenic compounds [6,100,101,102]. These chemicals may contribute to carcinogenesis through receptor-mediated signaling, metabolic reprogramming, adipose tissue inflammation, and epigenetic changes, as summarized in Figure 3.

Mechanistically, EDCs may contribute to hormone-dependent carcinogenesis through several interconnected pathways. First, estrogenic or anti-estrogenic compounds, including BPA, phthalates, dioxins, and PCBs, may mimic, interfere with, or block endogenous hormone signaling, particularly through estrogen receptors, thereby promoting abnormal proliferative responses in hormone-sensitive tissues [9,103]. In parallel, some EDCs may favor cellular metabolic reprogramming, a process that supports rapid cancer cell growth, survival, and progression [29,104]. These effects may be amplified by altered adipose tissue–tumor interactions, especially in metabolically active tissues, where EDC-related disruption of adipocyte function can promote a chronic pro-inflammatory phenotype [105,106]. This inflammatory microenvironment is accompanied by altered adipokine secretion, which may further stimulate tumor cell proliferation, invasion, and metastatic potential [107,108]. Finally, EDCs may induce epigenetic modifications, including changes in DNA methylation patterns, histone modifications, and noncoding RNA expression, thereby affecting gene expression and hormone receptor activity and contributing to oncogenic transformation [109,110]. Collectively, these pathways support a potential association between EDC exposure and hormone-dependent cancers, including testicular dysgenesis syndrome [111], ovarian [112,113], testicular [114], prostate [115], and thyroid cancers [116]. In addition to indirect endocrine-mediated effects, EDCs may directly modulate cancer cell physiology and function [6,110,117]. Some non-metabolizable EDCs can accumulate in adipose tissue lipid droplets and be progressively released, resulting in long-term, low-level systemic exposure [118,119]. However, the precise molecular mechanisms linking EDCs to hormone-dependent carcinogenesis remain incompletely understood [120], and causal inference is limited by mixture exposures, long latency, and heterogeneous exposure assessment, complicating the development of safer alternative products [121].

Early-life exposure represents a particularly vulnerable window, as EDCs may disrupt endocrine signaling during placental function, organogenesis, brain development, growth, metabolism, thyroid regulation, puberty, and reproductive maturation [122,123,124,125,126,127]. In children, EDCs have been associated with neurodevelopmental disorders through disruption of thyroid hormone signaling or metabolism [124], as well as with childhood obesity and disorders of sexual development [125]. Mechanistically, these effects may involve altered neuronal communication, neuronal growth and migration, and synaptic function [126]. Pubertal exposure may also increase susceptibility to breast cancer later in life [128] and has been linked to premature thelarche in female infants [129]. Despite increasing evidence regarding physical and neurodevelopmental effects, regulatory measures remain insufficient to fully limit EDC exposure from children’s food, personal care, and hygiene products [125].

#### 3.4.5. Immune System and Thyroid Hormone Dysfunction

EDCs may disrupt thyroid hormone synthesis, transport, metabolism, receptor signaling, and hypothalamic–pituitary–thyroid axis regulation, with pregnant women, fetuses, and infants being particularly vulnerable [130]. Sodium/iodide symporter inhibitors have been associated with altered thyroid function [131], while microplastics and microplastic-associated chemicals may interfere with multiple hormone receptors and hypothalamic axes, including the hypothalamic–pituitary–thyroid, hypothalamic–pituitary–adrenal, and hypothalamic–pituitary–gonadal axes [37,132]. Some EDCs may also act as androgenic or anti-androgenic agents and as agonists or antagonists of thyroid hormone receptors, further supporting their multi-axis endocrine activity [133].

Beyond thyroid disruption, EDCs may affect immune regulation, with particular relevance during pregnancy and fetal development [134]. Exposure has been associated with altered immune function and increased susceptibility to infection through immunosuppressive mechanisms [135]. Compounds such as BPA, phthalates, tetrachlorodibenzodioxin, propanil, triclosan, tributyltin, phenols, diethylstilbestrol, and parabens may influence immune cell development, function, survival, cytokine and immunoglobulin synthesis, inflammatory mediator production, and regulatory T-cell generation [136,137]. Depending on the compound, exposure window, and dose, these effects may contribute either to impaired host defense or to exaggerated inflammatory and allergic responses [138,139,140].

Experimental evidence also suggests that BPA may affect embryonic skeletal development by inducing apoptosis in bone precursor cells, further supporting the developmental sensitivity to endocrine-active compounds [138].

### 3.5. Reducing the Exposure and Exposure Effects of the Most Harmful Categories of EDC’s

To effectively reduce both exposure to EDCs and their associated health effects, it is essential to assess three key factors: (1) the EDC categories that pose the greatest risk to human health; (2) the biological and developmental factors that influence individual susceptibility to EDCs; and (3) strategies that can effectively minimize both exposure and the adverse effects of EDCs.

#### 3.5.1. Selected EDC Classes of Public Health Concern

Given the large number and heterogeneity of chemicals reported to have endocrine-active or endocrine-disrupting properties, this section focuses on selected EDC classes of public health concern rather than implying a definitive ranking of hazard across all substances. These classes were selected because they are frequently discussed in the literature in relation to human exposure, endocrine-related mechanisms, vulnerable populations, regulatory relevance, and potential health outcomes. Importantly, the strength of evidence and the extent to which endocrine disruption represents a critical effect differ across compounds, endpoints, exposure levels, and regulatory assessments [1,9,24,32,141,142,143,144,145,146,147,148]. The selected EDC categories, also represented in Figure 1, include:

(a) Bisphenols and phthalates are commonly found in plastics, food packaging, personal care products, and consumer materials. These compounds have been frequently investigated in relation to reproductive, metabolic, developmental, and neurodevelopmental outcomes, although interpretation depends on compound, dose, exposure window, and endpoint assessed [1,9,141,142].
(b) Dioxins and polychlorinated biphenyls (PCBs), many of which are classified as persistent organic pollutants, are characterized by environmental persistence, lipophilicity, and bioaccumulation. They have been discussed in relation to thyroid-related, reproductive, developmental, immune, and carcinogenic outcomes, but endocrine disruption may not represent the critical effect for all congeners or regulatory contexts [1,24,143,144].
(c) Per- and polyfluoroalkyl substances (PFAS) and pesticides represent heterogeneous chemical groups with diverse uses, exposure routes, and toxicological profiles. Some compounds within these groups have been evaluated for endocrine-related endpoints, including thyroid, reproductive, developmental, and metabolic outcomes; however, evidence strength and critical effects vary substantially across substances and assessments [141,145,146,147].
(d) Brominated flame retardants include compounds used in furniture, electronics, textiles, and other consumer products. Some members of this class have been investigated for thyroid, neurodevelopmental, reproductive, and metabolic effects, particularly in relation to indoor dust exposure and early-life vulnerability, although the endocrine relevance of critical effects differs by compound [1,148].

#### 3.5.2. Assessing Variations in Health Impact Following EDC Exposure

It is important to recognize that the health effects of EDC exposure vary among individuals due to multiple factors. These include differences in developmental windows of susceptibility [149], the complexity of EDC mixtures [150], and non-monotonic dose–response relationships [151], in which the magnitude of effect does not necessarily increase with dose. Additional factors influencing individual vulnerability include genetic variability [44], dietary habits, and modern lifestyle practices [152]. Moreover, the adverse effects of EDCs can be delayed [1], with some outcomes not manifesting for years or even decades [153], complicating the direct attribution of disease to specific exposures [21,154].

Some EDCs exhibit pronounced effects at low doses [21,146] while producing diminished or even opposite effects [155] at higher doses. This non-monotonic response complicates risk assessment and challenges the assumption that all exposures are inherently harmful. Therefore the relationship between the dose of an EDC and the resulting health effect can be complex [9] and in many cases response relationships can be non-monotonic, nonlinear and context-dependent.

Importantly, low-dose biological effects should not automatically be interpreted as adverse. Some responses may represent transient adaptive changes within physiological homeostatic limits, whereas others may become adverse when they are persistent, occur during critical developmental windows, affect hormone-dependent programming, or result in measurable functional impairment.

Therefore, the interpretation of low-dose effects requires consideration of the affected endocrine pathway, exposure timing, reversibility, target population, and downstream health outcome. Similarly, likely thresholds cannot be generalized across all EDCs.

For some compounds and endpoints, threshold-like behavior may exist, while for others, especially those acting through hormone receptors, developmental programming, epigenetic mechanisms, or mixtures, the dose–response relationship may be nonlinear or non-monotonic. This uncertainty supports a precautionary interpretation, particularly for fetuses, infants, children, pregnant women, and occupationally exposed populations.

Consequently, the relationship between EDC dose and health outcome can be complex, nonlinear, and many effects are subtle, further undermining the traditional toxicological principle that “the dose makes the poison,” [156] and making it difficult to fully evaluate the population-level impact of EDC exposure.

#### 3.5.3. Strategies to Reduce EDC Exposure and Mitigate Health Effects Post-Exposure

Although EDCs cannot be completely avoided or eliminated, a range of strategies is available to reduce exposure and mitigate potential health effects [157]. Their pervasive presence in food, water, air, and consumer products [158] makes total avoidance virtually impossible.

Despite increased public awareness and strengthened regulatory frameworks [159], EDCs remain detectable in the blood and tissues [160] of populations worldwide [50], including in pregnant women and newborns [161]. Consequently, effective public health strategies must address both the prevention of exposure and the reduction in biological and clinical effects once exposure has occurred.

Our proposed approach is based on two primary criteria: (1) prevention of exposure [1], and, if prevention fails, then (2) mitigation of the effects following exposure. These interventions must be tailored to different developmental stages, including prenatal, natal, childhood, and adulthood, and should be adapted according to the four most harmful EDC categories.

#### 3.5.4. Preventive and Exposure-Reduction Strategies for BPA and Phthalates

BPA and phthalates are extensively used in the manufacture of plastics, food packaging, cosmetics, and household products [162]. Preventive recommendations should be interpreted in relation to dose, frequency, duration, route of exposure, and likely exposure thresholds, rather than as implying that any contact with BPA or phthalates is necessarily harmful. For non-persistent compounds such as BPA and several phthalates, single or occasional low-level exposures may be rapidly metabolized and excreted, and their biological relevance depends on whether internal exposure reaches levels capable of producing measurable endocrine or toxicological effects [1].

Therefore, exposure-reduction strategies are most relevant when contact is repeated, cumulative, occurs through multiple sources, or involves vulnerable periods such as pregnancy, infancy, and childhood. In this context, the aim of prevention is not to imply that all real-life exposures are adverse, but to reduce avoidable and repeated exposures when safer alternatives are feasible.

Human exposure occurs primarily through ingestion of contaminated food and water [163], dermal absorption from personal care products, and handling of thermal paper receipts [164]. Consequently, preventive strategies should focus on reducing avoidable, repeated, or high-frequency contact with major exposure sources, particularly during pregnancy, infancy and childhood.

During the prenatal period, prevention involves behavioral and dietary modifications. Pregnant women are advised to avoid heating food in plastic containers [165], to use glass or ceramic alternatives, and to prefer fresh or frozen foods over canned products to reduce BPA ingestion from can linings.

The use of fragrance-free, phthalate-free personal care products and minimizing contact with thermal receipts further lowers exposure [166]. Healthcare providers should reinforce these measures through prenatal counseling and occupational risk assessment, particularly for individuals with frequent exposure to plastics or receipts [167].

During the natal period, preventive measures include selecting medical devices, feeding bottles, and toys that are free of phthalates [168]. Hospitals should prioritize the use of phthalate-free intravenous tubing and medical equipment, while parents are advised to avoid plastic feeding products labeled as “soft PVC” (Polyvinyl chloride).

In childhood, prevention emphasizes the home environment. Families should choose phthalate-free toys and furnishings, avoid microwaving food in plastic containers, and promote regular handwashing to reduce ingestion of contaminated dust.

In adulthood, preventive strategies focus on continued avoidance of PVC-containing materials, selection of fragrance-free personal care products, and minimizing contact with thermal receipts. These measures collectively contribute to reducing ongoing exposure to BPA and phthalates across the lifespan [169].

However, when preventive measures fail—which is common due to the pervasive presence of plastic-based materials—attention must shift to mitigating the effects of exposure [170]. Once absorbed, BPA and phthalates can disrupt reproductive and thyroid hormone signaling and alter metabolic regulation. In such cases, a diet rich in fiber and antioxidants may facilitate excretion and reduce oxidative stress [171].

In children and adults, additional chemical exposures, such as cigarette smoke—which has been associated with decreased thyroid-stimulating hormone levels and elevated triiodothyronine (T3) and thyroxine (T4) concentrations—should be avoided [172]. Healthcare providers may also monitor thyroid and reproductive hormone levels in exposed individuals to identify imbalances early and implement timely interventions.

#### 3.5.5. Preventive and Exposure-Reduction Strategies for Dioxins and Polychlorinated Biphenyls

Dioxins and polychlorinated biphenyls (PCBs) are persistent organic pollutants that bioaccumulate in the food chain, particularly in high-fat animal products [173]. Because these chemicals are lipophilic and highly resistant to environmental degradation [174], preventive strategies should prioritize dietary modifications and environmental exposure control.

During pregnancy, dietary modification constitutes the primary preventive strategy. Pregnant women are advised to limit the intake of fatty meats and high-fat dairy products [175], and to select smaller, low-trophic fish from verified, uncontaminated sources [176], which are less likely to contain elevated levels of dioxins or PCBs.

Nutritional counseling during prenatal visits should emphasize safe fish consumption in accordance with local advisories.

In the natal period, breastfeeding is strongly recommended despite the potential presence of trace contaminants, as its overall benefits outweigh the risks [177]. Maternal diet during lactation should continue to prioritize lean, uncontaminated food sources to minimize infant exposure.

During childhood, prevention emphasizes adherence to local food and soil contamination guidelines. Children should avoid fish or game from contaminated areas [178] and minimize contact with soil near industrial sites or waste disposal facilities [179]. Routine household cleaning using wet methods and HEPA-filtered vacuums can further reduce dust-borne contaminants [180].

In adulthood, similar dietary precautions remain important, and additional occupational safety measures are warranted for individuals working in waste incineration, recycling, or electrical equipment handling [181], where dioxin and PCB exposure may be elevated.

If preventive measures fail and dioxin or PCB exposure occurs, mitigation should focus on minimizing physiological effects through dietary management [182], clinical monitoring, and long-term metabolic support [183]. Because these chemicals bioaccumulate in adipose tissue, gradual weight management [184]—rather than rapid fat loss [185]—is recommended to avoid sudden mobilization of stored toxins into the bloodstream.

Diets rich in fiber, cruciferous vegetables, and whole grains may enhance the excretion of metabolites, while antioxidants and omega-3 fatty acids help modulate inflammatory pathways [175].

Clinicians should regularly monitor thyroid function, lipid metabolism, and reproductive health, as these systems are particularly susceptible to dioxin and PCB toxicity [186]. In affected communities, public health initiatives providing nutritional guidance and medical surveillance can play a critical role in mitigating long-term adverse effects.

#### 3.5.6. Preventive and Exposure-Reduction Strategies for per- and Polyfluoroalkyl Substances and Pesticides

Per- and polyfluoroalkyl substances (PFAS) and pesticides are characterized by environmental persistence and multiple exposure pathways [187]. PFAS are commonly found in nonstick cookware, water-repellent textiles, and firefighting foams [188], whereas pesticides are present in food residues, household products, and agricultural environments [189].

During the prenatal period, preventive strategies should prioritize access to clean water and uncontaminated food sources. Pregnant women are advised to use certified water filters capable of removing PFAS [190] and to avoid damaged or aged nonstick cookware [191,192].

Exposure to PFAS from indoor furniture can be minimized through intentional selection of materials with improved stain resistance that do not rely on PFAS-based finishes [193]. For pesticides, choosing organic produce, thoroughly washing and peeling fruits and vegetables, and diversifying dietary intake can help reduce cumulative exposure [194].

During the natal period, preventive measures should focus on maintaining a chemical-safe environment. Hospitals and families are advised to avoid PFAS-treated infant products [190] and to use natural materials for bedding and clothing. Home pest management should employ non-chemical or low-toxicity methods [195] whenever possible.

In childhood, exposure can be further reduced by selecting untreated carpets and clothing [196], ensuring adequate ventilation [197] in schools and homes, and adhering to safe pesticide application practices [198].

In adulthood, the same principles apply, with the addition of occupational safety measures for individuals working in high-risk environments where PFAS or pesticide exposure may be elevated.

When preventive measures fail and exposure to PFAS or pesticides occurs, the focus shifts to mitigating their effects on endocrine and metabolic health.

Transitioning to organic land management practices [199] represents a long-term strategy to reduce pesticide exposure, prevent soil and water contamination, and limit bioaccumulation of these toxicants within the food chain, benefiting both consumers and agricultural workers [200].

Nutritional support remains a primary defense mechanism. Diets rich in fiber, antioxidants, and chlorophyll-containing vegetables may facilitate the binding and excretion [201] of some chemicals [202].

Given that PFAS and certain pesticides can disrupt thyroid function and lipid metabolism [203], periodic monitoring of these parameters is essential. For women of reproductive age, fertility assessments and preconception counseling are recommended, as some PFAS compounds have been linked to alterations in reproductive hormone levels [204]. Community-level interventions, including blood testing programs and the provision of clean drinking water, are critical for mitigating cumulative, population-level effects of these EDCs [205].

#### 3.5.7. Preventive and Exposure-Reduction Strategies for Brominated Flame Retardants

Brominated flame retardants (BFRs), widely used in furniture, electronics, and textiles [206], can migrate into indoor dust and are readily ingested or inhaled, particularly by young children [207]. Preventive strategies therefore focus on the home environment and consumer product selection. Measures include frequent handwashing, regular household cleaning to reduce dust accumulation, and combined behavioral practices [208] aimed at minimizing contact with contaminated surfaces.

During the prenatal period, parents preparing nurseries should select furniture and mattresses free of added flame retardants [209], opting for certified natural or barrier-based materials [210]. Regular wet dusting and vacuuming [211] with HEPA filters [212] can substantially reduce indoor dust contamination.

In the natal and early infancy stages, untreated natural fabrics are recommended for infant clothing and bedding, and frequent handwashing should be encouraged [213]. During childhood, maintaining clean, dust-free play areas and avoiding heavily upholstered furniture are essential preventive measures [214]. In adulthood, careful handling of home renovations and disposal of treated foam or electronics is advised, as improper management of these materials can result in re-exposure to BFRs [215].

When exposure to brominated flame retardants has already occurred, physiological support should focus on mitigating oxidative stress and enhancing detoxification [216]. Given that BFRs are lipophilic and stored in adipose tissue [217], maintaining a healthy weight and balanced diet is essential [218]. Nutrients such as selenium, vitamin E, and polyphenols may help counteract the oxidative and inflammatory processes triggered by these chemicals [219]. Regular physical activity supports circulation and metabolic regulation [220], while minimizing further contact with dust or chemical pollutants prevents additional accumulation [221].

For infants and children, pediatric follow-up should include developmental monitoring, as BFR exposure has been linked to neurobehavioral and thyroid effects [222]. Adults with known exposure, particularly those employed in recycling or electronics industries, should undergo periodic medical screening to detect early thyroid or metabolic abnormalities [223].

## 4. Discussion

The evidence presented underscores that effective management of EDCs necessitates a dual-axis approach: prevention [1] prior to exposure and mitigation following exposure. These strategies should not be regarded as discrete or sequential stages, but rather as continuous and complementary components of endocrine health protection [147].

During the preventive phase, interventions focus on minimizing contact with contaminated food, air, water, and consumer products [224]. Such measures are largely behavioral and environmental, relying on informed decision-making at the household, community, and institutional levels [225].

However, given the global prevalence and environmental persistence of these chemicals, prevention alone cannot ensure complete safety. Consequently, post-exposure strategies—emphasizing physiological resilience, nutritional optimization, and clinical monitoring—are essential to mitigate the adverse effects of EDCs [226].

This integrated framework acknowledges the biological variability in vulnerability to EDCs across the life course. During pregnancy and infancy, the endocrine and metabolic systems are undergoing critical development, and even low-dose exposures can exert disproportionate effects. In this context, nutritional support, facilitation of detoxification pathways, and early clinical monitoring constitute the cornerstone of harm reduction [227].

In childhood, dietary and behavioral interventions enhance the body’s detoxification capacity, while active lifestyles and balanced nutrition help minimize bioaccumulation of EDCs [124]. By adulthood, preventive efforts must extend to long-term disease risk reduction, with regular clinical monitoring and maintenance of metabolic homeostasis serving as essential protective measures [228].

Importantly, mitigation strategies must also incorporate the psychosocial dimension of exposure. Awareness of EDC contamination can induce stress, anxiety, or feelings of helplessness among affected individuals [229].

Consequently, risk communication and psychological support should be integral components of any intervention program. Empowering individuals through nutrition, physical activity, and informed consumption converts awareness into agency and contributes to overall resilience [230].

From a public health perspective, this framework emphasizes the role of equity. Many preventive and post-exposure measures, such as acquiring organic foods, safer furnishings, or certified water filters, are influenced by socioeconomic status [231]. Accordingly, population-level interventions—including regulatory policies, subsidies for safer products, and community-level remediation programs—are critical to ensure equitable protection from EDCs across diverse populations [232].

The intersection of individual behavior and structural policy ultimately determines the effectiveness of any EDC reduction strategy. While prevention begins at the household level, recovery and resilience rely on coordinated healthcare systems, transparent industry practices, and consistent governmental regulation [169].

## 5. Limitations

This review provides a structured narrative synthesis and descriptive evidence mapping rather than a formal quantitative meta-analysis. A pooled quantitative analysis was not considered methodologically appropriate because the included studies differed substantially in EDC class, individual compounds, exposure biomarkers, biological matrices, timing and duration of exposure, study populations, health endpoints, and outcome definitions. In addition, many studies evaluated chemical mixtures rather than single compounds, while several clinically relevant outcomes may appear only after long latency periods or following exposure during vulnerable developmental windows. These factors limit direct numerical comparison across studies and complicate causal inference. Nevertheless, the descriptive evidence-mapping approach allowed us to identify EDC classes and outcome domains with stronger evidence coverage, as well as areas where data remain limited, inconsistent or emerging.

Another limitation is that not all included studies reported sex-disaggregated or gender-specific results. Therefore, sex-specific interpretation was provided when available, but some outcomes could only be discussed at the general population level.

## 6. Conclusions

The global presence of endocrine-disrupting chemicals is unlikely to be eliminated in the near future. Nevertheless, potential health risks may be reduced through a coherent framework that integrates exposure prevention, risk-based assessment, and targeted post-exposure monitoring across the life course. Importantly, the presence of an endocrine-active compound does not necessarily imply adverse health effects at all real-life exposure levels. The biological relevance of exposure depends on dose, frequency, duration, route of exposure, toxicokinetics, internal concentration, timing of exposure, and the vulnerability of the target population.

During the prenatal period, infancy, and childhood, prevention should prioritize the reduction in avoidable, repeated, or cumulative exposures, particularly when safer alternatives are feasible. However, low-dose biological responses should not automatically be interpreted as adverse. Some responses may be adaptive, transient, or reversible, whereas others may become harmful when they are persistent, occur during critical developmental windows, disrupt hormone-dependent programming, or lead to measurable functional impairment. Similarly, likely thresholds cannot be generalized across all EDCs, because they differ according to compound, endpoint, exposure route, target tissue, mixture context, and population susceptibility.

Therefore, a forward-looking strategy should combine reasonable exposure reduction with compound-specific risk assessment, biomonitoring when appropriate, clinical surveillance of vulnerable groups, transparent risk communication, and regulatory thresholds where available. Prevention without consideration of dose may overstate risk, whereas mitigation without exposure reduction may be insufficient for persistent or cumulative exposures. Sustainable protection against endocrine disruption should therefore rely on balanced, evidence-based strategies that integrate toxicological thresholds, developmental vulnerability, individual behavior, clinical monitoring, and public health policy.

## Figures and Tables

**Table 1:** Main EDC classes, target populations, mechanisms, and associated health outcomes.

EDC Class	Representative Compounds	Main Target Populations/Settings	Main Mechanisms/Adverse Pathways	Main Reported Health Outcomes
**Bisphenols and phthalates**	Bisphenol A, alternative bisphenols, DEHP, DBP and other phthalates	Pregnant women, children, reproductive-age women and men, general population exposed through plastics, food packaging, cosmetics, and personal care products	Estrogen receptor modulation, anti-androgenic effects, altered steroidogenesis, thyroid receptor interference, epigenetic changes, oxidative stress, PPARγ activation, altered adipogenesis	Female reproductive dysfunction, reduced oocyte quality, endometriosis, polycystic ovary syndrome, altered puberty, adverse pregnancy outcomes, reduced semen quality, DNA damage in spermatozoa, obesity, insulin resistance, type 2 diabetes, thyroid disruption
**Dioxins and PCBs**	TCDD, dioxin-like PCBs, non-dioxin-like PCBs	Pregnant women, fetuses, children, adults, occupationally or accidentally exposed populations	Aryl hydrocarbon receptor activation, anti-estrogenic effects, altered steroidogenesis, thyroid hormone transport and metabolism disruption, oxidative stress, immune modulation, bioaccumulation in adipose tissue	Neurodevelopmental impairment, thyroid dysfunction, reproductive toxicity, altered fertility, metabolic disorders, diabetes risk, immune dysfunction, hormone-sensitive cancers
**Organochlorine pesticides and other pesticides**	DDT, endosulfan, lindane, chlordane, dieldrin, atrazine, organophosphates, carbamates, pyrethroids	Agricultural workers, occupationally exposed adults, residents in agricultural areas, pregnant women, children exposed through food residues or contaminated environments	Estrogenic, anti-estrogenic, androgenic and anti-androgenic activity, AHR activation, altered cytochrome P450 activity, disruption of hypothalamic–pituitary–gonadal and thyroid axes, mitochondrial dysfunction, oxidative stress	Male and female infertility, altered semen parameters, ovarian dysfunction, adverse pregnancy outcomes, obesity, metabolic disorders, thyroid dysfunction, hormone-sensitive cancers
**PFAS**	PFOA, PFOS and related compounds	General population exposed through contaminated water, food, non-stick cookware, treated textiles and indoor environments; pregnant women and children	Thyroid hormone disruption, altered lipid metabolism, immune modulation, oxidative stress, interference with reproductive hormone signaling	Thyroid dysfunction, dyslipidemia, metabolic alterations, reproductive hormone changes, developmental effects, immune-related effects
**Brominated flame retardants**	PBDEs, HBCDD, TBBPA	Infants and children exposed through indoor dust, pregnant women, electronics/recycling workers, residents exposed to treated furniture and textiles	Thyroid hormone disruption, neuroendocrine interference, oxidative stress, developmental toxicity, bioaccumulation	Neurodevelopmental effects, thyroid dysfunction, altered growth and development, reproductive effects, metabolic alterations
**Heavy metals**	Cadmium, lead, mercury, arsenic	Children, pregnant women, adults with contaminated food/water exposure, occupationally exposed populations	Oxidative stress, interference with steroidogenesis, receptor signaling disruption, epigenetic changes, testicular and ovarian toxicity	Developmental toxicity, reproductive dysfunction, testicular damage, thyroid/metabolic effects, cardiovascular risk
**Pharmaceuticals, nonylphenols and personal care-related EDCs**	Diethylstilbestrol, triclosan, parabens, nonylphenols, selected pharmaceuticals	Women, men, children, consumers exposed through personal care products, detergents, industrial oils, and pharmaceutical residues	Estrogenic or anti-estrogenic activity, altered hormone metabolism, receptor-mediated signaling, antimicrobial-related endocrine and immune effects	Reproductive dysfunction, developmental abnormalities, hormone-sensitive outcomes, possible thyroid and immune effects
**Microplastics**	Plastic particles containing additives or adsorbed pollutants	General population, children, pregnant women; exposure through food, water, air, and indoor dust	Potential vector or source of bisphenols, phthalates, flame retardants, pesticides, POPs, metals and other adsorbed contaminants; oxidative stress and inflammation	Emerging evidence; potential endocrine, metabolic, reproductive, thyroid, immune, and developmental effects, depending on particle composition and associated chemicals

AHR, aryl hydrocarbon receptor; DBP, dibutyl phthalate; DEHP, di(2-ethylhexyl) phthalate; EDCs, endocrine-disrupting chemicals; HBCDD, hexabromocyclododecane; PCBs, polychlorinated biphenyls; PFAS, per- and polyfluoroalkyl substances; PFOA, perfluorooctanoic acid; PFOS, perfluorooctane sulfonate; POPs, persistent organic pollutants; PPARγ, peroxisome proliferator-activated receptor gamma; PBDEs, polybrominated diphenyl ethers; TBBPA, tetrabromobisphenol A; TCDD, 2,3,7,8-tetrachlorodibenzo-p-dioxin.

**Table 2:** Descriptive evidence mapping of the literature included in the review according to EDC class and main outcome domain.

Evidence Domain	Number of Sources Identified in the Final Synthesis	Most Frequent Type of Evidence	Main Outcomes/Mechanisms Covered	Interpretation of Evidence Coverage
Bisphenols and phthalates	21	Human biomonitoring studies, epidemiological studies, experimental studies, mechanistic reviews, systematic reviews/meta-analyses	Reproductive dysfunction, altered steroidogenesis, pregnancy outcomes, obesity, insulin resistance, type 2 diabetes, thyroid disruption, developmental outcomes, hormone-sensitive cancers	High evidence coverage; findings are relatively consistent for reproductive and metabolic outcomes, although causality remains limited by observational designs, continuous low-level exposure, and co-exposure to other chemicals
Dioxins, PCBs, and persistent organic pollutants	18	Epidemiological studies, accidental/occupational exposure studies, food-chain exposure studies, toxicological models, reviews	Thyroid dysfunction, neurodevelopmental outcomes, reproductive toxicity, immune dysfunction, metabolic disorders, cardiovascular risk, cancer-related endpoints	High evidence coverage; persistence, lipophilicity, bioaccumulation, placental/lactational transfer, and long latency support concern for long-term and developmental effects
Organochlorine pesticides and other pesticides	11	Occupational and residential exposure studies, biomonitoring studies, experimental models, reviews	Male and female fertility, altered semen parameters, ovarian dysfunction, thyroid disruption, metabolic disorders, hormone-sensitive cancers, developmental outcomes	Moderate-to-high evidence coverage; stronger evidence exists for organochlorine pesticides, whereas findings for other pesticide classes are more heterogeneous
PFAS	10	Biomonitoring studies, cohort and cross-sectional studies, exposure-reduction studies, reviews and guidance documents	Thyroid dysfunction, lipid metabolism, immune effects, reproductive hormone changes, developmental outcomes, exposure reduction	Moderate evidence coverage; findings are suggestive but vary according to individual PFAS compound, exposure metric, biological matrix, and target population
Brominated flame retardants	12	Indoor exposure studies, biomonitoring studies, developmental and toxicological studies, occupational/recycling exposure studies, reviews	Thyroid disruption, neurodevelopmental outcomes, growth and developmental effects, reproductive effects, metabolic alterations, oxidative stress	Moderate evidence coverage; children, indoor dust exposure, and occupational exposure are particularly relevant, but exposure assessment remains heterogeneous
Heavy metals	5	Epidemiological, occupational, biomonitoring, and mechanistic studies	Reproductive toxicity, developmental toxicity, thyroid/metabolic effects, cardiovascular risk, food contamination	Moderate evidence coverage; endocrine effects are biologically plausible but often coexist with broader toxic effects
Pharmaceuticals, nonylphenols, triclosan, parabens, and personal care-related EDCs	8	Experimental studies, biomonitoring studies, intervention studies, selected epidemiological studies, reviews	Estrogenic/anti-estrogenic effects, reproductive outcomes, hormone-sensitive effects, thyroid and immune effects, exposure reduction	Low-to-moderate evidence coverage; evidence differs substantially across compounds and exposure settings
Microplastics and microplastic-associated chemicals	2	Emerging exposure studies and narrative/environmental reviews	Potential vector or source of endocrine-active additives and adsorbed pollutants; possible endocrine, metabolic, reproductive, thyroid, immune, and developmental effects	Emerging evidence; interpretation depends on polymer composition, additives, adsorbed contaminants, particle size, and exposure route
Reproductive and fertility outcomes	35	Human epidemiological studies, fertility studies, experimental studies, systematic reviews/meta-analyses	Male fertility, semen quality, sperm DNA damage, ovarian function, oocyte quality, puberty, pregnancy outcomes, hormone-sensitive reproductive disorders	Highest outcome-specific evidence coverage in the present synthesis
Metabolic outcomes, obesity, diabetes, liver and lipid metabolism	22	Epidemiological studies, experimental studies, systematic reviews/meta-analyses, mechanistic reviews	Obesity, adipogenesis, insulin resistance, type 2 diabetes, NAFLD, lipid metabolism, mitochondrial dysfunction, oxidative stress	High evidence coverage, especially for bisphenols, phthalates, POPs, PCBs, pesticides, PFAS, and selected metals
Developmental, neurodevelopmental, and child-health outcomes	30	Cohort studies, developmental toxicology studies, child-health reviews, mechanistic studies	Prenatal growth, neurodevelopment, thyroid-mediated developmental effects, puberty, childhood obesity, immune and metabolic programming	High evidence coverage; prenatal life, infancy, and childhood are repeatedly identified as vulnerable windows
Thyroid, adrenal, and hypothalamic–pituitary axis outcomes	11	Mechanistic studies, systematic reviews, endocrine-axis reviews, biomonitoring studies	Thyroid hormone synthesis, transport, metabolism, receptor signaling, NIS inhibition, adrenal steroidogenesis, hypothalamic–pituitary axis disruption	Moderate evidence coverage; evidence is biologically plausible but varies by compound and exposure window
Cancer-related outcomes	17	Mechanistic studies, cancer-focused reviews, systematic reviews/meta-analyses	Breast, ovarian, testicular, prostate, thyroid cancers, testicular dysgenesis syndrome, epigenetic and metabolic reprogramming	Moderate-to-high evidence coverage; causal inference remains limited by long latency and mixture exposure
Cardiovascular outcomes	8	Epidemiological studies, systematic reviews/meta-analyses, mechanistic studies	Cardiovascular mortality, hypertension, endothelial dysfunction, atherosclerosis, cardiometabolic risk	Moderate evidence coverage, mostly for metals, POPs, BPA, phthalates, and pesticide-related metabolites
Immune and inflammatory outcomes	5	Mechanistic reviews, immunotoxicology studies, pregnancy-related immune studies	Immunosuppression, altered cytokines, regulatory T-cell effects, allergic/inflammatory responses, autoimmune-related effects	Lower evidence coverage compared with reproductive, metabolic, developmental, and thyroid outcomes

Counts are descriptive and refer to the sources included in the final reference list of this review. Because several sources addressed more than one EDC class, mechanism, exposure pathway, or health outcome, some references were counted in more than one category. Therefore, the numbers should be interpreted as evidence mapping rather than as a formal bibliometric, systematic, or meta-analytic quantification.

## Data Availability

No new data were created or analyzed in this study. Data sharing is not applicable to this article.

## Abbreviations

The following abbreviations are used in this manuscript:

EDCs	Endocrine-disrupting chemicals
BPA	Bisphenol A
PFAS	Per- and polyfluoroalkyl substances
PCBs	Polychlorinated biphenyls
BFRs	Brominated flame retardants
PVC	Polyvinyl chloride
HEPA	High-efficiency particulate air
DNA	Deoxyribonucleic acid
RNA	Ribonucleic acid
ATP	Adenosine triphosphate
T3	Triiodothyronine
T4	Thyroxine
NHANES	National Health and Nutrition Examination Survey
MEDLINE	Medical Literature Analysis and Retrieval System Online
BPA-free	Bisphenol A-free
PFAS-treated	Treated with per- and polyfluoroalkyl substances
Phthalate-free	Free of phthalates

## References

[1] Yilmaz B., Terekeci H., Sandal S., Kelestimur F. (2020). Endocrine disrupting chemicals: Exposure, effects on human health, mechanism of action, models for testing and strategies for prevention. Rev. Endocr. Metab. Disord..

[2] Vos J.G., Dybing E., Greim H.A., Ladefoged O., Lambré C., Tarazona J.V., Brandt I., Vethaak A.D. (2000). Health effects of endocrine-disrupting chemicals on wildlife, with special reference to the European situation. Crit. Rev. Toxicol..

[3] Zlatnik M.G. (2016). Endocrine-Disrupting Chemicals and Reproductive Health. J. Midwifery Women’s Health.

[4] Braun J.M. (2017). Early-life exposure to EDCs: Role in childhood obesity and neurodevelopment. Nat. Rev. Endocrinol..

[5] Kirkley A.G., Sargis R.M. (2014). Environmental endocrine disruption of energy metabolism and cardiovascular risk. Curr. Diab Rep..

[6] Modica R., Benevento E., Colao A. (2023). Endocrine-disrupting chemicals (EDCs) and cancer: New perspectives on an old relationship. J. Endocrinol. Investig..

[7] Lobstein T., Brownell K.D. (2021). Endocrine-disrupting chemicals and obesity risk: A review of recommendations for obesity prevention policies. Obes. Rev..

[8] Lisco G., Giagulli V.A., Iovino M., Guastamacchia E., Pergola G., Triggiani V. (2022). Endocrine-Disrupting Chemicals: Introduction to the Theme. Endocr. Metab. Immune Disord. Drug Targets.

[9] Anne B., Raphael R., Feingold K.R., Ahmed S.F., Anawalt B., Blackman M.R., Chrousos G., Corpas E., de Herder W.W., Dhatariya K., Hamilton E., Hofland J. (2000). Endocrine Disruptor Chemicals. Endotext.

[10] La Merrill M.A., Vandenberg L.N., Smith M.T., Goodson W., Browne P., Patisaul H.B., Guyton K.Z., Kortenkamp A., Cogliano V.J., Woodruff T.J. (2020). Consensus on the key characteristics of endocrine-disrupting chemicals as a basis for hazard identification. Nat. Rev. Endocrinol..

[11] David S., Amandine A. (2025). Endocrine-disrupting effects of contaminants on communication and behaviors of insects: From molecular effects to ecological consequences. Curr. Opin. Insect Sci..

[12] Lanphear B.P., Hornung R., Khoury J., Yolton K., Baghurst P., Bellinger D.C., Canfield R.L., Dietrich K.N., Bornschein R., Greene T. (2005). Low-level environmental lead exposure and children’s intellectual function: An international pooled analysis. Environ. Health Perspect..

[13] Buttke D.E., Sircar K., Martin C. (2012). Exposures to endocrine-disrupting chemicals and age of menarche in adolescent girls in NHANES (2003–2008). Environ. Health Perspect..

[14] Endocrine Society (2023). Endocrine-Disrupting Chemicals in the European Union. https://www.endocrine.org/advocacy/position-statements/endocrine-disrupting-chemicals-in-the-european-union.

[15] Xin F., Susiarjo M., Bartolomei M.S. (2015). Multigenerational and transgenerational effects of endocrine disrupting chemicals: A role for altered epigenetic regulation?. Semin. Cell Dev. Biol..

[16] Brehm E., Flaws J.A. (2019). Transgenerational Effects of Endocrine-Disrupting Chemicals on Male and Female Reproduction. Endocrinology.

[17] Greenspan L.C., Lee M.M. (2018). Endocrine disrupters and pubertal timing. Curr. Opin. Endocrinol. Diabetes Obes..

[18] Ho V., Pelland-St-Pierre L., Gravel S., Bouchard M.F., Verner M.-A., Labrèche F. (2022). Endocrine disruptors: Challenges and future directions in epidemiologic research. Environ. Res..

[19] Vandenberg L.N. (2014). Low-dose effects of hormones and endocrine disruptors. Vitam. Horm..

[20] Rhomberg L.R., Goodman J.E. (2012). Low-dose effects and nonmonotonic dose-responses of endocrine disrupting chemicals: Has the case been made?. Regul. Toxicol. Pharmacol..

[21] Vandenberg L.N., Colborn T., Hayes T.B., Heindel J.J., Jacobs D.R., Lee D.-H., Shioda T., Soto A.M., vom Saal F.S., Welshons W.V. (2012). Hormones and endocrine-disrupting chemicals: Low-dose effects and nonmonotonic dose responses. Endocr. Rev..

[22] Welshons W.V., Thayer K.A., Judy B.M., Taylor J.A., Curran E.M., vom Saal F.S. (2003). Large effects from small exposures. I. Mechanisms for endocrine-disrupting chemicals with estrogenic activity. Environ. Health Perspect..

[23] Hayes T.B., Anderson L.L., Beasley V.R., de Solla S.R., Iguchi T., Ingraham H., Kestemont P., Kniewald J., Kniewald Z., Langlois V.S. (2011). Demasculinization and feminization of male gonads by atrazine: Consistent effects across vertebrate classes. J. Steroid Biochem. Mol. Biol..

[24] Encarnação T., Pais A.A., Campos M.G., Burrows H.D. (2019). Endocrine disrupting chemicals: Impact on human health, wildlife and the environment. Sci. Prog..

[25] Tyagi P., James-Todd T., Mínguez-Alarcón L., Ford J.B., Keller M., Petrozza J., Calafat A.M., Hauser R., Williams P.L., Bellavia A. (2021). Identifying windows of susceptibility to endocrine disrupting chemicals in relation to gestational weight gain among pregnant women attending a fertility clinic. Environ. Res..

[26] Calkins K., Devaskar S.U. (2011). Fetal origins of adult disease. Curr. Probl. Pediatr. Adolesc. Health Care.

[27] Harding J.E. (2001). The nutritional basis of the fetal origins of adult disease. Int. J. Epidemiol..

[28] Wadhwa P.D., Buss C., Entringer S., Swanson J.M. (2009). Developmental origins of health and disease: Brief history of the approach and current focus on epigenetic mechanisms. Semin. Reprod. Med..

[29] Papalou O., Kandaraki E.A., Papadakis G., Diamanti-Kandarakis E. (2019). Endocrine Disrupting Chemicals: An Occult Mediator of Metabolic Disease. Front. Endocrinol..

[30] Liang Y., Lu Q., Chen M., Zhao X., Chu C., Zhang C., Yuan J., Liu H., Lash G.E. (2025). Impact of endocrine disrupting chemicals (EDCs) on epigenetic regulation in the uterus: A narrative review. Reprod. Biol. Endocrinol..

[31] Amato A.A., Wheeler H.B., Blumberg B. (2021). Obesity and endocrine-disrupting chemicals. Endocr. Connect..

[32] Ledesma K.N.Z., Hernández M.A., Chávez K.R., Barajas A.F.A., Vázquez D.P.A., Santiago G.G., Castro A.A., Barrera T.D.R. (2025). Endocrine Disruptors and Their Impact on Quality of Life: A Literature Review. Cureus.

[33] Matos D.M., Ramos J., Brandão A., Baeta A., Rodrigues I., dos Santos I., Coentro J., Fernandes J., de Carvalho L.B., Marques M. (2024). Microplastics ingestion and endocrine disrupting chemicals (EDCs) by breeding seabirds in the east tropical Atlantic: Associations with trophic and foraging proxies (δ15N and δ13C). Sci. Total Environ..

[34] Peivasteh-Roudsari L., Barzegar-Bafrouei R., Sharifi K.A., Azimisalim S., Karami M., Abedinzadeh S., Asadinezhad S., Tajdar-Oranj B., Mahdavi V., Alizadeh A.M. (2023). Origin, dietary exposure, and toxicity of endocrine-disrupting food chemical contaminants: A comprehensive review. Heliyon.

[35] Plunk E.C., Richards S.M. (2020). Endocrine-Disrupting Air Pollutants and Their Effects on the Hypothalamus-Pituitary-Gonadal Axis. Int. J. Mol. Sci..

[36] Annamalai J., Namasivayam V. (2015). Endocrine disrupting chemicals in the atmosphere: Their effects on humans and wildlife. Environ. Int..

[37] Mogus J.P., Marin M., Arowolo O., Salemme V., Suvorov A. (2024). Developmental exposures to common environmental pollutants result in long-term Reprogramming of hypothalamic-pituitary axis in mice. Environ. Pollut..

[38] Anderson S.E., Meade B.J. (2014). Potential health effects associated with dermal exposure to occupational chemicals. Environ. Health Insights.

[39] Ju Q., Zouboulis C.C. (2016). Endocrine-disrupting chemicals and skin manifestations. Rev. Endocr. Metab. Disord..

[40] Skinner M. (2016). Epigenetic transgenerational inheritance. Nat. Rev. Endocrinol..

[41] Shafei A., Ramzy M.M., Hegazy A.I., Husseny A.K., El-Hadary U.G., Taha M.M., Mosa A.A. (2018). The molecular mechanisms of action of the endocrine disrupting chemical bisphenol A in the development of cancer. Gene.

[42] Shanle E.K., Xu W. (2011). Endocrine disrupting chemicals targeting estrogen receptor signaling: Identification and mechanisms of action. Chem. Res. Toxicol..

[43] Li Y., Luh C.J., Burns K.A., Arao Y., Jiang Z., Teng C.T., Tice R.R., Korach K.S. (2013). Endocrine-Disrupting Chemicals (EDCs): In Vitro Mechanism of Estrogenic Activation and Differential Effects on ER Target Genes. Environ. Health Perspect..

[44] Wallis D.J., Truong L., La Du J., Tanguay R.L., Reif D.M. (2021). Uncovering Evidence for Endocrine-Disrupting Chemicals That Elicit Differential Susceptibility through Gene-Environment Interactions. Toxics.

[45] Lee H.R., Jeung E.B., Cho M.H., Kim T.H., Leung P.C., Choi K.C. (2013). Molecular mechanism(s) of endocrine-disrupting chemicals and their potent oestrogenicity in diverse cells and tissues that express oestrogen receptors. J. Cell Mol. Med..

[46] Combarnous Y., Nguyen T.M.D. (2019). Comparative Overview of the Mechanisms of Action of Hormones and Endocrine Disruptor Compounds. Toxics.

[47] Stepulak A., Rzeski W., Sifringer M., Brocke K., Gratopp A., Kupisz K., Turski L., Ikonomidou C. (2008). Fluoxetine inhibits the extracellular signal regulated kinase pathway and suppresses growth of cancer cells. Cancer Biol. Ther..

[48] Ahn C., Jeung E.-B. (2023). Endocrine-Disrupting Chemicals and Disease Endpoints. Int. J. Mol. Sci..

[49] Fitz-James M.H., Cavalli G. (2022). Molecular mechanisms of transgenerational epigenetic inheritance. Nat. Rev. Genet..

[50] Endocrine Society (2023). Common EDCs and Where They Are Found. https://www.endocrine.org/topics/edc/what-edcs-are/common-edcs.

[51] Alavian-Ghavanini A., Rüegg J. (2017). Understanding Epigenetic Effects of Endocrine Disrupting Chemicals: From Mechanisms to Novel Test Methods. Basic. Clin. Pharmacol. Toxicol..

[52] Manikkam M., Tracey R., Guerrero-Bosagna C., Skinner M.K. (2013). Plastics derived endocrine disruptors (BPA, DEHP and DBP) induce epigenetic transgenerational inheritance of obesity, reproductive disease and sperm epimutations. PLoS ONE.

[53] Cheong A., Zhang X., Cheung Y.-Y., Tang W.-Y., Chen J., Ye S.-H., Medvedovic M., Leung Y.-K., Prins G.S., Ho S.-M. (2016). DNA methylome changes by estradiol benzoate and bisphenol A links early-life environmental exposures to prostate cancer risk. Epigenetics.

[54] Zhao M., Niu Y., Huang Q., Li W. (2025). Exploring the Mechanisms of EDCs-Induced Metabolic Disorders in Humans Using Network Toxicology and Molecular Docking. NAM J..

[55] Jaskulak M., Zimowska M., Rolbiecka M., Zorena K. (2025). Understanding the role of endocrine disrupting chemicals as environmental obesogens in the obesity epidemic: A comprehensive overview of epidemiological studies between 2014 and 2024. Ecotoxicol. Environ. Saf..

[56] Janesick A.S., Blumberg B. (2016). Obesogens: An emerging threat to public health. Am. J. Obstet. Gynecol..

[57] Lee H.K., Shim E.B. (2013). Extension of the mitochondria dysfunction hypothesis of metabolic syndrome to atherosclerosis with emphasis on the endocrine-disrupting chemicals and biophysical laws. J. Diabetes Investig..

[58] Boix-Castejón M., Roche E., Olivares-Vicente M., Álvarez-Martínez F.J., Herranz-López M., Micol V. (2023). Plant compounds for obesity treatment through neuroendocrine regulation of hunger: A systematic review. Phytomedicine.

[59] Furukawa S., Fujita T., Shimabukuro M., Iwaki M., Yamada Y., Nakajima Y., Nakayama O., Makishima M., Matsuda M., Shimomura I. (2004). Increased oxidative stress in obesity and its impact on metabolic syndrome. J. Clin. Investig..

[60] Gálvez-Ontiveros Y., Páez S., Monteagudo C., Rivas A. (2020). Endocrine Disruptors in Food: Impact on Gut Microbiota and Metabolic Diseases. Nutrients.

[61] Francis C.E., Allee L., Nguyen H., Grindstaff R.D., Miller C.N., Rayalam S. (2021). Endocrine disrupting chemicals: Friend or foe to brown and beige adipose tissue?. Toxicology.

[62] The Institute for Functional Medicine (2025). Endocrine-Disrupting Chemicals and Type 2 Diabetes. https://www.ifm.org/articles/endocrine-disruptors-type-2-diabetes.

[63] Dagar M., Kumari P., Mirza A.M.W., Singh S., Ain N.U., Munir Z., Javed T., Virk M.F.I., Javed S., Qizilbash F.H. (2023). The Hidden Threat: Endocrine Disruptors and Their Impact on Insulin Resistance. Cureus.

[64] Schulz M.C., Sargis R.M. (2021). Inappropriately sweet: Environmental endocrine-disrupting chemicals and the diabetes pandemic. Adv. Pharmacol..

[65] Aayush M.V., Nikthesh G., Rajmohan D., Ravindran C.P., Vasantharekha R., Thangavelu S., Seetharaman B. (2025). Redefining the pathogenesis of Gestational Diabetes Mellitus: The cumulative impact of endocrine disrupting environmental chemicals in key metabolic pathways. Med. Hypotheses.

[66] Haverinen E., Fernandez M.F., Mustieles V., Tolonen H. (2021). Metabolic Syndrome and Endocrine Disrupting Chemicals: An Overview of Exposure and Health Effects. Int. J. Environ. Res. Public Health.

[67] Küblbeck J., Vuorio T., Niskanen J., Fortino V., Braeuning A., Abass K., Rautio A., Hakkola J., Honkakoski P., Levonen A.-L. (2020). The EDCMET Project: Metabolic Effects of Endocrine Disruptors. Int. J. Mol. Sci..

[68] Pan K., Xu J., Xu Y., Wang C., Yu J. (2024). The association between endocrine disrupting chemicals and nonalcoholic fatty liver disease: A systematic review and meta-analysis. Pharmacol. Res..

[69] Le Magueresse-Battistoni B. (2021). Endocrine disrupting chemicals and metabolic disorders in the liver: What if we also looked at the female side?. Chemosphere.

[70] Maradonna F., Carnevali O. (2018). Lipid Metabolism Alteration by Endocrine Disruptors in Animal Models: An Overview. Front. Endocrinol..

[71] Fan Y., Tao C., Li Z., Huang Y., Yan W., Zhao S., Gao B., Xu Q., Qin Y., Wang X. (2023). Association of Endocrine-Disrupting Chemicals with All-Cause and Cause-Specific Mortality in the U.S.: A Prospective Cohort Study. Environ. Sci. Technol..

[72] Guo X., Li N., Wang H., Su W., Song Q., Liang Q., Liang M., Sun C., Li Y., Lowe S. (2022). Combined exposure to multiple metals on cardiovascular disease in NHANES under five statistical models. Environ. Res..

[73] Fenercioglu A.K., Unal D.O. (2025). The Role of Endocrine Disrupting Chemicals in the Development of Atherosclerosis. Cardiovasc. Toxicol..

[74] Lin T.A., Zhou C. (2025). Epigenetic impact of endocrine-disrupting chemicals on atherosclerosis. Essays Biochem..

[75] Fu X., Xu J., Zhang R., Yu J. (2020). The association between environmental endocrine disruptors and cardiovascular diseases: A systematic review and meta-analysis. Environ. Res..

[76] Carpenter D.O. (2006). Polychlorinated biphenyls (PCBs): Routes of exposure and effects on human health. Rev. Environ. Health.

[77] Rubin B.S. (2011). Bisphenol A: An endocrine disruptor with widespread exposure and multiple effects. J. Steroid Biochem. Mol. Biol..

[78] Meeker J.D., Sathyanarayana S., Swan S.H. (2009). Phthalates and other additives in plastics: Human exposure and associated health outcomes. Philos. Trans. R. Soc. Lond. B Biol. Sci..

[79] Newbold R.R. (2004). Lessons learned from perinatal exposure to diethylstilbestrol. Toxicol. Appl. Pharmacol..

[80] Park S.H., Lim J.E., Park H., Jee S.H. (2016). Body burden of persistent organic pollutants on hypertension: A meta-analysis. Environ. Sci. Pollut. Res. Int..

[81] Wu W., Ziglioli F., Maestroni U. (2020). Male Reproductive Health.

[82] Marques-Pinto A., Carvalho D. (2013). Human infertility: Are endocrine disruptors to blame?. Endocr. Connect..

[83] Inoshita H., Masuyama H., Hiramatsu Y. (2003). The different effects of endocrine-disrupting chemicals on estrogen receptor-mediated transcription through interaction with coactivator TRAP220 in uterine tissue. J. Mol. Endocrinol..

[84] Lahimer M., Diwan M.A., Montjean D., Cabry R., Bach V., Ajina M., Ben Ali H., Benkhalifa M., Khorsi-Cauet H. (2023). Endocrine disrupting chemicals and male fertility: From physiological to molecular effects. Front. Public Health.

[85] Henriques M.C., Loureiro S., Fardilha M., Herdeiro M.T. (2019). The role of endocrine-disrupting Chemicals in Male Fertility Decline. Male Reproductive Health.

[86] Dobrzyńska M.M., Radzikowska J. (2013). Genotoxicity and reproductive toxicity of bisphenol A and X-ray/bisphenol A combination in male mice. Drug Chem. Toxicol..

[87] Tiwari D., Vanage G. (2013). Mutagenic effect of Bisphenol A on adult rat male germ cells and their fertility. Reprod. Toxicol..

[88] El-Beshbishy H.A., Aly H.A., El-Shafey M. (2013). Lipoic acid mitigates bisphenol A-induced testicular mitochondrial toxicity in rats. Toxicol. Ind. Health.

[89] Rahman M.S., Kwon W.S., Lee J.S., Yoon S.J., Ryu B.Y., Pang M.G. (2015). Bisphenol-A affects male fertility via fertility-related proteins in spermatozoa. Sci. Rep..

[90] Harper A.P., Finger B.J., Green M.P. (2020). Chronic Atrazine Exposure Beginning Prenatally Impacts Liver Function and Sperm Concentration With Multi-Generational Consequences in Mice. Front. Endocrinol..

[91] Tricotteaux-Zarqaoui S., Lahimer M., Diwan M.A., Corona A., Candela P., Cabry R., Bach V., Khorsi-Cauet H., Benkhalifa M. (2024). Endocrine disruptor chemicals exposure and female fertility declining: From pathophysiology to epigenetic risks. Front. Public Health.

[92] Hassan S., Thacharodi A., Priya A., Meenatchi R., Hegde T.A., R T., Nguyen H., Pugazhendhi A. (2024). Endocrine disruptors: Unravelling the link between chemical exposure and Women’s reproductive health. Environ. Res..

[93] Crain D.A., Janssen S.J., Edwards T.M., Heindel J., Ho S.-M., Hunt P., Iguchi T., Juul A., McLachlan J.A., Schwartz J. (2008). Female reproductive disorders: The roles of endocrine-disrupting compounds and developmental timing. Fertil. Steril..

[94] Cao L.-L., Yan C.-H., Yu X.-D., Tian Y., Zhao L., Liu J.-X., Shen X.-M. (2011). Relationship between serum concentrations of polychlorinated biphenyls and organochlorine pesticides and dietary habits of pregnant women in Shanghai. Sci. Total Environ..

[95] Zama A.M., Bhurke M.A., Uzumcu M. (2016). Effects of Endocrine-disrupting Chemicals on Female Reproductive Health. Open Biotechnol. J..

[96] Iguchi T., Takasugi N. (1986). Polyovular follicles in the ovary of immature mice exposed prenatally to diethylstilbestrol. Anat. Embryol..

[97] Suzuki A., Sugihara A., Uchida K., Sato T., Ohta Y., Katsu Y., Watanabe H., Iguchi T. (2002). Developmental effects of perinatal exposure to bisphenol-A and diethylstilbestrol on reproductive organs in female mice. Reprod. Toxicol..

[98] Hunt P.A., Sathyanarayana S., Fowler P.A., Trasande L. (2016). Female Reproductive Disorders, Diseases, and Costs of Exposure to Endocrine Disrupting Chemicals in the European Union. J. Clin. Endocrinol. Metab..

[99] Chiaffarino F., Parazzini F., La Vecchia C., Chatenoud L., Di Cintio E., Marsico S. (1999). Diet and uterine myomas. Obstet. Gynecol..

[100] Dogan S., Simsek T. (2016). Possible relationship between endocrine disrupting chemicals and hormone dependent gynecologic cancers. Med. Hypotheses.

[101] Rutkowska A.Z., Szybiak A., Serkies K., Rachoń D. (2016). Endocrine disrupting chemicals as potential risk factor for estrogen-dependent cancers. Pol. Arch. Med. Wewn..

[102] Buoso E., Masi M., Racchi M., Corsini E. (2020). Endocrine-Disrupting Chemicals’ (EDCs) Effects on Tumour Microenvironment and Cancer Progression: Emerging Contribution of RACK1. Int. J. Mol. Sci..

[103] Khan N.G., Correia J., Adiga D., Rai P.S., Dsouza H.S., Chakrabarty S., Kabekkodu S.P. (2021). A comprehensive review on the carcinogenic potential of bisphenol A: Clues and evidence. Environ. Sci. Pollut. Res. Int..

[104] Winz C., Zong W.X., Suh N. (2023). Endocrine-disrupting compounds and metabolomic reprogramming in breast cancer. J. Biochem. Mol. Toxicol..

[105] Boudalia S., Bousbia A., Boumaaza B., Oudir M., Canivenc Lavier M.C. (2021). Relationship between endocrine disruptors and obesity with a focus on bisphenol A: A narrative review. Bioimpacts.

[106] Bateman M.E., Strong A.L., McLachlan J.A., Burow M.E., Bunnell B.A. (2017). The Effects of Endocrine Disruptors on Adipogenesis and Osteogenesis in Mesenchymal Stem Cells: A Review. Front. Endocrinol..

[107] Soto A.M., Sonnenschein C. (2010). Environmental causes of cancer: Endocrine disruptors as carcinogens. Nat. Rev. Endocrinol..

[108] Umar M.I., Hassan W., Murtaza G., Buabeid M., Arafa E., Irfan H.M., Asmawi M.Z., Huang X. (2021). The Adipokine Component in the Molecular Regulation of Cancer Cell Survival, Proliferation and Metastasis. Pathol. Oncol. Res..

[109] Singh D.D. (2025). Epigenetic Mechanisms of Endocrine-Disrupting Chemicals in Breast Cancer and Their Impact on Dietary Intake. J. Xenobiotics.

[110] Akanbi C.A., Rotimi D.E., Ojo A.B., Ojo O.A. (2025). Endocrine-disrupting chemicals (EDCs) and epigenetic regulation in embryonic development: Mechanisms, impacts, and emerging trends. Toxicol. Rep..

[111] Xing J.S., Bai Z.M. (2018). Is testicular dysgenesis syndrome a genetic, endocrine, or environmental disease, or an unexplained reproductive disorder?. Life Sci..

[112] Macedo S., Teixeira E., Gaspar T.B., Boaventura P., Soares M.A., Miranda-Alves L., Soares P. (2023). Endocrine-disrupting chemicals and endocrine neoplasia: A forty-year systematic review. Environ. Res..

[113] Pan J., Liu P., Yu X., Zhang Z., Liu J. (2024). The adverse role of endocrine disrupting chemicals in the reproductive system. Front. Endocrinol..

[114] Bräuner E.V., Lim Y.-H., Koch T., Uldbjerg C.S., Gregersen L.S., Pedersen M.K., Frederiksen H., Petersen J.H., A Coull B., Andersson A.-M. (2021). Endocrine Disrupting Chemicals and Risk of Testicular Cancer: A Systematic Review and Meta-analysis. J. Clin. Endocrinol. Metab..

[115] Corti M., Lorenzetti S., Ubaldi A., Zilli R., Marcoccia D. (2022). Endocrine Disruptors and Prostate Cancer. Int. J. Mol. Sci..

[116] Alsen M., Sinclair C., Cooke P., Ziadkhanpour K., Genden E., van Gerwen M. (2021). Endocrine Disrupting Chemicals and Thyroid Cancer: An Overview. Toxics.

[117] Guarnotta V., Amodei R., Frasca F., Aversa A., Giordano C. (2022). Impact of Chemical Endocrine Disruptors and Hormone Modulators on the Endocrine System. Int. J. Mol. Sci..

[118] Bokobza E., Hinault C., Tiroille V., Clavel S., Bost F., Chevalier N. (2021). The Adipose Tissue at the Crosstalk Between EDCs and Cancer Development. Front. Endocrinol..

[119] Veiga-Lopez A., Pu Y., Gingrich J., Padmanabhan V. (2018). Obesogenic Endocrine Disrupting Chemicals: Identifying Knowledge Gaps. Trends Endocrinol. Metab..

[120] Zhou J.X., Chen S.S., Zheng Z.Y., Yuan W.B., Liu X.B., Ni H.G. (2025). Mechanisms of three typical endocrine-disrupting chemicals causing myocardial infarction: Gene-level computational modeling. J. Environ. Chem. Ecotoxicol..

[121] Rochefort H. (2017). Endocrine disruptors (EDs) and hormone-dependent cancers: Correlation or causal relationship?. Comptes Rendus Biol..

[122] Meeker J.D. (2012). Exposure to environmental endocrine disruptors and child development. Arch. Pediatr. Adolesc. Med..

[123] Bali D., Scaltrito F., Grimaldi M.T., Giardino I., Pettoello-Mantovani M., Pastore M. (2023). The impact of the endocrine disruptors on child health. Glob. Pediatr..

[124] Di Pietro G., Forcucci F., Chiarelli F. (2023). Endocrine Disruptor Chemicals and Children’s Health. Int. J. Mol. Sci..

[125] The Lancet Child & Adolescent Health (2024). EDCs: A threat to child health. Lancet Child. Adolesc. Health.

[126] Schug T.T., Blawas A.M., Gray K., Heindel J.J., Lawler C.P. (2015). Elucidating the links between endocrine disruptors and neurodevelopment. Endocrinology.

[127] Predieri B., Iughetti L., Bernasconi S., Street M.E. (2022). Endocrine Disrupting Chemicals’ Effects in Children: What We Know and What We Need to Learn?. Int. J. Mol. Sci..

[128] Lucaccioni L., Trevisani V., Marrozzini L., Bertoncelli N., Predieri B., Lugli L., Berardi A., Iughetti L. (2020). Endocrine-Disrupting Chemicals and Their Effects during Female Puberty: A Review of Current Evidence. Int. J. Mol. Sci..

[129] Scsukova S., Rollerova E., Bujnakova Mlynarcikova A. (2016). Impact of endocrine disrupting chemicals on onset and development of female reproductive disorders and hormone-related cancer. Reprod. Biol..

[130] Pearce E.N. (2024). Endocrine Disruptors and Thyroid Health. Endocr. Pract..

[131] Arriagada A.A., Albornoz E., Opazo M.C., Becerra A., Vidal G., Fardella C., Michea L., Carrasco N., Simon F., Elorza A.A. (2015). Excess iodide induces an acute inhibition of the sodium/iodide symporter in thyroid male rat cells by increasing reactive oxygen species. Endocrinology.

[132] Jang H., Calder L., Choi J.W., Kwon B.R., Pearce E.N., Shin H.M. (2025). Associations between exposure to sodium/iodide symporter inhibitors and markers of thyroid function: A systematic review and meta-analysis. Chemosphere.

[133] Diamanti-Kandarakis E., Bourguignon J.-P., Giudice L.C., Hauser R., Prins G.S., Soto A.M., Zoeller R.T., Gore A.C. (2009). Endocrine-disrupting chemicals: An Endocrine Society scientific statement. Endocr. Rev..

[134] Schjenken J.E., Green E.S., Overduin T.S., Mah C.Y., Russell D.L., Robertson S.A. (2021). Endocrine Disruptor Compounds-A Cause of Impaired Immune Tolerance Driving Inflammatory Disorders of Pregnancy?. Front. Endocrinol..

[135] Bansal A., Henao-Mejia J., Simmons R.A. (2018). Immune System: An Emerging Player in Mediating Effects of Endocrine Disruptors on Metabolic Health. Endocrinology.

[136] Huang R.-G., Li X.-B., Wang Y.-Y., Wu H., Li K.-D., Jin X., Du Y.-J., Wang H., Qian F.-Y., Li B.-Z. (2023). Endocrine-disrupting chemicals and autoimmune diseases. Environ. Res..

[137] Nowak K., Jabłońska E., Ratajczak-Wrona W. (2019). Immunomodulatory effects of synthetic endocrine disrupting chemicals on the development and functions of human immune cells. Environ. Int..

[138] Chanemougavally J., Thotakura B., Shruthy K.M., Janaki C.S., Chanemougavally J. (2023). Effects of Endocrine Disrupting Chemicals (EDCs) on Skeletal System Development: A Review. Cureus.

[139] Kuo C.H., Yang S.N., Kuo P.L., Hung C.H. (2012). Immunomodulatory effects of environmental endocrine disrupting chemicals. Kaohsiung J. Med. Sci..

[140] Chalubinski M., Kowalski M.L. (2006). Endocrine disrupters—Potential modulators of the immune system and allergic response. Allergy.

[141] Hilz E.N., Gore A.C. (2023). Endocrine-Disrupting Chemicals: Science and Policy. Policy Insights Behav. Brain Sci..

[142] Ghassabian A., Vandenberg L., Kannan K., Trasande L. (2022). Endocrine-Disrupting Chemicals and Child Health. Annu. Rev. Pharmacol. Toxicol..

[143] Kumar M., Sarma D.K., Shubham S., Kumawat M., Verma V., Prakash A., Tiwari R. (2020). Environmental Endocrine-Disrupting Chemical Exposure: Role in Non-Communicable Diseases. Front. Public Health.

[144] Kahn L.G., Philippat C., Nakayama S.F., Slama R., Trasande L. (2020). Endocrine-disrupting chemicals: Implications for human health. Lancet Diabetes Endocrinol..

[145] Leung Y.K. (2023). A Silent Threat: Exploring the Impact of Endocrine Disruption on Human Health. Int. J. Mol. Sci..

[146] Lind T., Dunder L., Lejonklou M.H., Lind P.M., Melhus H., Lind L. (2025). Developmental low-dose bisphenol A exposure leads to extensive transcriptome female masculinization and male feminization later in life. Commun. Med..

[147] Klingelhöfer D., Braun M., Dröge J., Brüggmann D., Groneberg D.A. (2025). Global research on endocrine disruptors as emerging hazards for human health and the environment. Front. Endocrinol..

[148] Mita D.G. (2016). Endocrine Disruptors: A Real Concern for Humans?. Open Biotechnol. J..

[149] Wright R.O. (2017). Environment, susceptibility windows, development, and child health. Curr. Opin. Pediatr..

[150] Ribeiro E., Ladeira C., Viegas S. (2017). EDCs Mixtures: A Stealthy Hazard for Human Health?. Toxics.

[151] Conolly R.B., Lutz W.K. (2004). Nonmonotonic dose-response relationships: Mechanistic basis, kinetic modeling, and implications for risk assessment. Toxicol. Sci..

[152] Balu U.R., Vasantharekha R., Paromita C., Ali K., Mudgal G., Kesari K.K., Seetharaman B. (2024). Linking EDC-laden food consumption and modern lifestyle habits with preeclampsia: A non-animal approach to identifying early diagnostic biomarkers through biochemical alterations. Food Chem. Toxicol..

[153] Heindel J.J., Vandenberg L.N. (2015). Developmental origins of health and disease: A paradigm for understanding disease cause and prevention. Curr. Opin. Pediatr..

[154] Toft G., Liew Z. (2022). Health Effects Associated with Exposures to Endocrine Disrupting Chemicals. Toxics.

[155] Stiefel C., Stintzing F. (2023). Endocrine-active and endocrine-disrupting compounds in food—Occurrence, formation and relevance. NFS J..

[156] Chen L., Giesy J.P., Xie P. (2018). The dose makes the poison. Sci. Total Environ..

[157] NIEHS (2024). Endocrine Disruptors. https://www.niehs.nih.gov/health/topics/agents/endocrine.

[158] Tripathi D., Singh S., Ahamad K.U. (2025). Understanding the plastic-associated endocrine-disrupting chemicals in India: Environmental contamination, health impacts and regulatory challenges. J. Environ. Chem. Eng..

[159] EDC FREE EUROPE (2024). Seven Priorities to Protect People and Environment from Endocrine-Disrupting Chemicals. https://www.edc-free-europe.org/articles/position-papers/seven-priorities-to-protect-people-and-environment-from-endocrine-disrupting-chemicals.

[160] Metcalfe C.D., Bayen S., Desrosiers M., Muñoz G., Sauvé S., Yargeau V. (2022). An introduction to the sources, fate, occurrence and effects of endocrine disrupting chemicals released into the environment. Environ. Res..

[161] Kelley A.S., Banker M., Goodrich J.M., Dolinoy D.C., Burant C., Domino S.E., Smith Y.R., Song P.X.K., Padmanabhan V. (2018). Early pregnancy exposure to endocrine disrupting chemical mixtures are associated with inflammatory changes in maternal and neonatal circulation. Sci. Rep..

[162] Warner G.R., Flaws J.A. (2018). Bisphenol A and Phthalates: How Environmental Chemicals Are Reshaping Toxicology. Toxicol. Sci..

[163] Carnevali O., Giorgini E., Canuti D., Mylonas C.C., Forner-Piquer I., Maradonna F. (2019). Diets contaminated with Bisphenol A and Di-isononyl phtalate modify skeletal muscle composition: A new target for environmental pollutant action. Sci. Total Environ..

[164] Bernier M.R., Vandenberg L.N. (2017). Handling of thermal paper: Implications for dermal exposure to bisphenol A and its alternatives. PLoS ONE.

[165] Alharbi M.H., Mumena W.A., Hammouda S.A. (2020). Use of Plastics with Hot Food among Saudi Pregnant Women Is Associated with Increased Concentrations of A1C, Thyroid-Stimulating Hormone, and Homocysteine and Decreased Concentrations of Vitamins and Minerals. Nutrients.

[166] Harley K.G., Kogut K., Madrigal D.S., Cardenas M., Vera I.A., Meza-Alfaro G., She J., Gavin Q., Zahedi R., Bradman A. (2016). Reducing Phthalate, Paraben, and Phenol Exposure from Personal Care Products in Adolescent Girls: Findings from the HERMOSA Intervention Study. Environ. Health Perspect..

[167] Trasande L., Sargis R.M. (2024). Endocrine-disrupting chemicals: Mainstream recognition of health effects and implications for the practicing internist. J. Intern. Med..

[168] Kim J.H., Kwak J.M., Kang H. (2021). Web-based behavioral intervention to reduce exposure to phthalate metabolites, bisphenol A, triclosan, and parabens in mothers with young children: A randomized controlled trial. Int. J. Hyg. Environ. Health.

[169] Martin L., Zhang Y., First O., Mustieles V., Dodson R., Rosa G., Coburn-Sanderson A., Adams C.D., Messerlian C. (2022). Lifestyle interventions to reduce endocrine-disrupting phthalate and phenol exposures among reproductive age men and women: A review and future steps. Environ. Int..

[170] Luo R., Zhang T., Wang L., Feng Y. (2023). Emissions and mitigation potential of endocrine disruptors during outdoor exercise: Fate, transport, and implications for human health. Environ. Res..

[171] Madore M.P., Sakaki J.R., Chun O.K. (2022). Protective effects of polyphenols against endocrine disrupting chemicals. Food Sci. Biotechnol..

[172] Babić Leko M., Gunjača I., Pleić N., Zemunik T. (2021). Environmental Factors Affecting Thyroid-Stimulating Hormone and Thyroid Hormone Levels. Int. J. Mol. Sci..

[173] Rahul C.M., Gayathri K., Kesavachandran C.N. (2024). Global trends of dioxin and dioxin-like PCBs in animal-origin foods: A systematic review and gap areas. Environ. Monit. Assess..

[174] Tahir E., Cordier S., Courtemanche Y., Forget-Dubois N., Desrochers-Couture M., Bélanger R.E., Ayotte P., Jacobson J.L., Jacobson S.W., Muckle G. (2020). Effects of polychlorinated biphenyls exposure on physical growth from birth to childhood and adolescence: A prospective cohort study. Environ. Res..

[175] Institute of Medicine (US) Committee on the Implications of Dioxin in the Food Supply (2003). Dioxins and Dioxin-like Compounds in the Food Supply: Strategies to Decrease Exposure.

[176] Mikołajczyk S., Warenik-Bany M., Maszewski S., Pajurek M. (2020). Farmed Fish as a Source of Dioxins and PCBs for Polish Consumers. J. Vet. Res..

[177] Korrick S.A., Altshul L. (1998). High breast milk levels of polychlorinated biphenyls (PCBs) among four women living adjacent to a PCB-contaminated waste site. Environ. Health Perspect..

[178] Judd N., Griffith W.C., Faustman E.M. (2004). Contribution of PCB exposure from fish consumption to total dioxin-like dietary exposure. Regul. Toxicol. Pharmacol..

[179] Baron C.P., Børresen T., Jacobsen C. (2007). Comparison of methods to reduce dioxin and polychlorinated biphenyls contents in fishmeal: Extraction and enzymatic treatments. J. Agric. Food Chem..

[180] Li H., Chen Y., Crittenden J., Hand D., Taylor R. (2006). Modeling of indoor air treatment of polychlorinated dibenzo-p-dioxins and dibenzofurans using high-efficiency particulate air-carbon filtration. J. Air Waste Manag. Assoc..

[181] Muzembo B.A., Iwai-Shimada M., Isobe T., Arisawa K., Shima M., Fukushima T., Nakayama S.F. (2019). Dioxins levels in human blood after implementation of measures against dioxin exposure in Japan. Environ. Health Prev. Med..

[182] Jin W., Otake M., Eguchi A., Sakurai K., Nakaoka H., Watanabe M., Todaka E., Mori C. (2017). Dietary Habits and Cooking Methods Could Reduce Avoidable Exposure to PCBs in Maternal and Cord Sera. Sci. Rep..

[183] Zabik M.E., Zabik M.J. (1999). Polychlorinated biphenyls, polybrominated biphenyls, and dioxin reduction during processing/cooking food. Adv. Exp. Med. Biol..

[184] Mochida Y., Fukata H., Matsuno Y., Mori C. (2007). Reduction of dioxins and polychlorinated biphenyls (PCBs) in human body. Fukuoka Igaku Zasshi.

[185] Kim M.J., Marchand P., Henegar C., Antignac J.-P., Alili R., Poitou C., Bouillot J.-L., Basdevant A., Le Bizec B., Barouki R. (2011). Fate and complex pathogenic effects of dioxins and polychlorinated biphenyls in obese subjects before and after drastic weight loss. Environ. Health Perspect..

[186] Leijs M.M., ten Tusscher G.W., Olie K., van Teunenbroek T., van Aalderen W.M., de Voogt P., Vulsma T., Bartonova A., von Krauss M.K., Mosoiu C. (2012). Thyroid hormone metabolism and environmental chemical exposure. Environ. Health.

[187] Food Safety Commission of Japan (2025). Per- and Poly-fluoroalkyl Substances (PFAS) (Chemicals and Contaminants). Food Saf..

[188] NIEHS (2025). Perfluoroalkyl and Polyfluoroalkyl Substances (PFAS). https://www.niehs.nih.gov/health/topics/agents/pfc.

[189] Ramesh A., Balasubramanian M. (1999). The impact of household preparations on the residues of pesticides in selected agricultural food commodities available in India. J. AOAC Int..

[190] National Academies of Sciences, Engineering, and Medicine, Health and Medicine Division, Division on Earth and Life Studies, Board on Population Health and Public Health Practice, Board on Environmental Studies and Toxicology, Committee on the Guidance on PFAS Testing and Health Outcomes (2022). Guidance on PFAS Exposure, Testing, and Clinical Follow-Up. 4 PFAS Exposure Reduction.

[191] Papini M.P., Senofonte M., Cuzzola R.A., Remmani R., Pettiti I., Riccardi C., Simonetti G. (2024). Adsorption Technology for PFAS Removal in Water: Comparison between Novel Carbonaceous Materials. Materials.

[192] Lee T., Speth T.F., Nadagouda M.N. (2022). High-pressure membrane filtration processes for separation of Per- and polyfluoroalkyl substances (PFAS). Chem. Eng. J..

[193] LaPier J., Blum A., Brown B.R., Kwiatkowski C.F., Phillips B., Ray H., Sun G. (2023). Evaluating the Performance of Per- and Polyfluoroalkyl Substance Finishes on Upholstery Fabrics. AATCC J. Res..

[194] Yang S.-J., Mun S., Kim H.J., Han S.J., Kim D.W., Cho B.-S., Kim A.G., Park D.W. (2022). Effectiveness of Different Washing Strategies on Pesticide Residue Removal: The First Comparative Study on Leafy Vegetables. Foods.

[195] Srivani Maddala V.K. (2019). Green pest management practices for sustainable buildings: Critical review. Sci. Prog..

[196] Yiin L.M., Lioy P.J., Rhoads G.G. (2003). Impact of home carpets on childhood lead intervention study. Environ. Res..

[197] Lipinski T., Ahmad D., Serey N., Jouhara H. (2020). Review of ventilation strategies to reduce the risk of disease transmission in high occupancy buildings. Int. J. Thermofluids.

[198] Oshingbade O.S., Moda H.M., Akinsete S.J., Adejumo M., Hassan N. (2025). Determinants of Safe Pesticide Handling and Application Among Rural Farmers. Int. J. Environ. Res. Public Health.

[199] Pesaresi P., Loit E. (2024). Editorial: Options for transition of land towards intensive and sustainable agricultural systems, volume II. Front. Plant Sci..

[200] Millán R., Schröder P., Sæbø A. (2019). Editorial: Options for Transition of Land Towards Intensive and Sustainable Agricultural Systems. Front. Plant Sci..

[201] Martins T., Barros A.N., Rosa E., Antunes L. (2023). Enhancing Health Benefits through Chlorophylls and Chlorophyll-Rich Agro-Food: A Comprehensive Review. Molecules.

[202] Lykkebo C.A., Nguyen K.H., Niklas A.A., Laursen M.F., Bahl M.I., Licht T.R., Mortensen M.S. (2024). Diet rich in soluble dietary fibres increases excretion of perfluorooctane sulfonic acid (PFOS) in male Sprague-Dawley rats. Food Chem. Toxicol..

[203] Coperchini F., Croce L., Ricci G., Magri F., Rotondi M., Imbriani M., Chiovato L. (2021). Thyroid Disrupting Effects of Old and New Generation PFAS. Front. Endocrinol..

[204] Xia Z., Chen S., Liu Y., Li J., Liu X., Zhang L., Xiang Q., Wu Y. (2025). In utero exposure to per- and polyfluoroalkyl substances and neonatal sex hormone levels: Implications of endocrine disrupting effects during critical development windows. J. Environ. Chem. Ecotoxicol..

[205] National Academies of Sciences, Engineering, and Medicine, Health and Medicine Division, Division on Earth and Life Studies, Board on Population Health and Public Health Practice, Board on Environmental Studies and Toxicology, Committee on the Guidance on PFAS Testing and Health Outcomes (2022). Guidance on PFAS Exposure, Testing, and Clinical Follow-Up. Appendix E, White Paper: Review of the PFAS Personal Intervention Literature.

[206] Morf L.S., Tremp J., Gloor R., Huber Y., Stengele M., Zennegg M. (2005). Brominated flame retardants in waste electrical and electronic equipment: Substance flows in a recycling plant. Environ. Sci. Technol..

[207] Hoffman K., Tang X., Cooper E.M., Hammel S.C., Sjodin A., Phillips A.L., Webster T.F., Stapleton H.M. (2024). Children’s exposure to brominated flame retardants in the home: The TESIE study. Environ. Pollut..

[208] Gibson E.A., Stapleton H.M., Calero L., Holmes D., Burke K., Martinez R., Cortes B., Nematollahi A., Evans D., Herbstman J.B. (2019). Flame retardant exposure assessment: Findings from a behavioral intervention study. J. Expo. Sci. Environ. Epidemiol..

[209] Betts K.S. (2008). New thinking on flame retardants. Environ. Health Perspect..

[210] Lucas D., Petty S.M., Keen O., Luedeka B., Schlummer M., Weber R., Barlaz M., Yazdani R., Riise B., Rhodes J. (2018). Methods of Responsibly Managing End-of-Life Foams and Plastics Containing Flame Retardants: Part I. Environ. Eng. Sci..

[211] National Research Council (US) Subcommittee on Flame-Retardant Chemicals (2000). Toxicological Risks of Selected Flame-Retardant Chemicals. 2 Assessment of Health Risks from the Use of Flame Retardants.

[212] Chen C.F., Hsu C.H., Chang Y.J., Lee C.H., Lee D.L. (2022). Efficacy of HEPA Air Cleaner on Improving Indoor Particulate Matter 2.5 Concentration. Int. J. Environ. Res. Public Health.

[213] Piérard G.E., Arrese J.E., Dowlati A., Daskaleros P.A., Rodriguez C. (1994). Effects of softened and unsoftened fabrics on infant skin. Int. J. Dermatol..

[214] Wilson J.M., Platts-Mills T.A.E. (2018). Home Environmental Interventions for House Dust Mite. J. Allergy Clin. Immunol. Pract..

[215] Nussbaum G.F. (2008). Alternative Waste Management Strategies. Perioper. Nurs. Clin..

[216] Li X., Liao J., Chen Z., Wang H., Long L. (2025). Oxidative stress mediates the association between brominated flame retardants and hyperlipidemia in US adults. Int. J. Environ. Health Res..

[217] Vail G.M., Walley S.N., Yasrebi A., Maeng A., Conde K.M., Roepke T.A. (2020). The interactions of diet-induced obesity and organophosphate flame retardant exposure on energy homeostasis in adult male and female mice. J. Toxicol. Environ. Health A.

[218] Knutsen H.K., Kvalem H.E., Thomsen C., Frøshaug M., Haugen M., Becher G., Alexander J., Meltzer H.M. (2008). Dietary exposure to brominated flame retardants correlates with male blood levels in a selected group of Norwegians with a wide range of seafood consumption. Mol. Nutr. Food Res..

[219] Han F., Xu R., Wang H., Gao X., Guo M. (2025). Tea Polyphenols Mitigate TBBPA-Induced Renal Injury Through Modulation of ROS-PI3K/AKT-NF-κB Signalling in Carp (*Cyprinus carpio*). Animals.

[220] Liu C., Hou H. (2023). Physical exercise and persistent organic pollutants. Heliyon.

[221] Zota A.R., Singla V., Adamkiewicz G., Mitro S.D., Dodson R.E. (2017). Reducing chemical exposures at home: Opportunities for action. J. Epidemiol. Community Health.

[222] Lyche J.L., Rosseland C., Berge G., Polder A. (2015). Human health risk associated with brominated flame-retardants (BFRs). Environ. Int..

[223] Stone M.B., Wallace R.B., Institute of Medicine (US) Committee on Medicare Coverage of Routine Thyroid Screening (2003). Medicare Coverage of Routine Screening for Thyroid Dysfunction. Appendix B, Screening for Thyroid Disease: Systematic Evidence Review.

[224] Srivastava R., Singh Y., White J.C., Dhankher O.P. (2024). Mitigating toxic metals contamination in foods: Bridging knowledge gaps for addressing food safety. Trends Food Sci. Technol..

[225] Yu S. (2024). Formation, Occurrence and Mitigation Strategies of Food Contaminants and Natural Toxicants: Challenges and Prospects. Foods.

[226] Corbett G.A., Lee S., Woodruff T.J., Hanson M., Hod M., Charlesworth A.M., Giudice L., Conry J., McAuliffe F.M., International Federation of Gynecology and Obstetrics (FIGO) Committee on Impact of Pregnancy on Long-term Health and the FIGO Committee on Climate Change and Toxic Environmental Exposures (2022). Nutritional interventions to ameliorate the effect of endocrine disruptors on human reproductive health: A semi-structured review from FIGO. Int. J. Gynaecol. Obstet..

[227] Ning J., Akhter T., Sarfraz M., Afridi H.I., Albasher G., Unar A. (2023). The importance of monitoring endocrine-disrupting chemicals and essential elements in biological samples of fertilizer industry workers. Environ. Res..

[228] Coman L.I., Ianculescu M., Paraschiv E.A., Alexandru A., Bădărău I.A. (2024). Smart Solutions for Diet-Related Disease Management: Connected Care, Remote Health Monitoring Systems, and Integrated Insights for Advanced Evaluation. Appl. Sci..

[229] Okman E., Yalçın S.S. (2024). Awareness and Knowledge of Endocrine-Disrupting Chemicals Among Pregnant Women and New Mothers: A Cross-Sectional Survey Study. Toxics.

[230] Leipold B., Klier K., Dapperger E., Schmidt A. (2024). Physical activity and nutrition in relation to resilience: A cross-sectional study. Sci. Rep..

[231] Vandenberg L.N., Rayasam S.D.G., Axelrad D.A., Bennett D.H., Brown P., Carignan C.C., Chartres N., Diamond M.L., Joglekar R., Shamasunder B. (2023). Addressing systemic problems with exposure assessments to protect the public’s health. Environ. Health.

[232] Seligman H.K., Berkowitz S.A. (2019). Aligning Programs and Policies to Support Food Security and Public Health Goals in the United States. Annu. Rev. Public Health.

